# Biologically informed deep learning for explainable epigenetic clocks

**DOI:** 10.1038/s41598-023-50495-5

**Published:** 2024-01-15

**Authors:** Aurel Prosz, Orsolya Pipek, Judit Börcsök, Gergely Palla, Zoltan Szallasi, Sandor Spisak, István Csabai

**Affiliations:** 1 Danish Cancer Institute, Copenhagen, Denmark; 2 Department of Physics of Complex Systems, ELTE Eötvös Loránd University, Budapest, Hungary; 3 Biotech Research & Innovation Centre (BRIC), University of Copenhagen, Copenhagen, Denmark; 4 Department of Biological Physics, ELTE Eötvös Loránd University, Budapest, Hungary; 5 Health Services Management Training Centre, Semmelweis University, Budapest, Hungary; 6 Institute of Enzymology, HUN-REN Research Centre for Natural Sciences, Budapest, Hungary

**Keywords:** Ageing, Machine learning

## Abstract

Ageing is often characterised by progressive accumulation of damage, and it is one of the most important risk factors for chronic disease development. Epigenetic mechanisms including DNA methylation could functionally contribute to organismal aging, however the key functions and biological processes may govern ageing are still not understood. Although age predictors called epigenetic clocks can accurately estimate the biological age of an individual based on cellular DNA methylation, their models have limited ability to explain the prediction algorithm behind and underlying key biological processes controlling ageing. Here we present XAI-AGE, a biologically informed, explainable deep neural network model for accurate biological age prediction across multiple tissue types. We show that XAI-AGE outperforms the first-generation age predictors and achieves similar results to deep learning-based models, while opening up the possibility to infer biologically meaningful insights of the activity of pathways and other abstract biological processes directly from the model.

## Introduction

Aging, defined as some form of functional decline over time, has always attracted a considerable interest among humankind, and has been in the focus of intense research from a wide range of perspectives^1^. According to the related studies, certain biomarkers can rather precisely predict the functional capability of tissues, organs and even patients^2,3^. Furthermore, age-related biomarkers enable the introduction of the concept of biological age^4,5^, which can bring additional information in the risk assessments for age-related conditions on top of chronological age.

One of the most promising age-predictive biomarkers are the ones based on DNA-methylation^6–8^, which can be used for basically any source of DNA from sorted cells through tissues to organs.

Age-related changes in DNA methylomes are generally occurring processes, during which up to 2–14% of all cytosine-guanine dinucleotide (CpG) sites display consistent changes in their methylation levels throughout ageing^9–18^.

The combination of multiple CpGs or even individual CpG sites are often used to estimate the chronologial age of cells, tissues, or individuals based on their DNA methylation levels, and are generally referred to as epigenetic age estimators or epigenetic clocks. The obtained estimated age is often referred to as DNAm age, or epigenetic age^8^, which is highly correlated with chronological age, but also affected by other biological factors^13,19–22^ such as health status.

Typically developed using supervised machine learning methods, DNA methylation-based age estimators often employ penalized regression models. These models are designed to autonomously identify the CpGs that are most informative for estimating age^8,23^. However, the construction of a multi-tissue DNA methylation based age estimator is non-trivial, due to the significant differences between different tissues^19,20^ and the distinct biological processes that drive the observed age-related hypermethylation and hypomethylation. The first multi-tissue DNA methylation-based age estimator became widely known as Horvath’s clock^6^ (proposed by Steve Horvath), which relied on elastic net regression that selected altogether 353 CpGs from the overall 27k CpG dinucleotides in the data it was trained on, corresponding to about 8,000 microarray samples collected from patients of all ages between children and elderly. Aside some limitations^24,25^, Horvath’s clock proved to be a remarkably accurate age estimator in a variety of studies, yielding precise results for diverse DNA sources spanning the whole human lifespan^8^, e.g., together with other similar DNA-methylation-based clocks^26–28^, Horvath’s clock was used to quantify the effectiveness of a program designed to regenerate the thymus, where the mean epigenetic age was 1.5 years younger than baseline after one year of treatment^29^. Possible relations between epigenetic aging and the previously identified aging hallmarks are in the focus of on going research, and very recent related results have shown that although epigenetic aging is distinct from genomic instability, cellular senescence and telomere attrition, it is associated with nutrient sensing, mitochondrial activity and stem cell composition^30^.

With the advent of the overwhelming success of neural network-based techniques and deep learning methods in pattern recognition problems in general, it became another natural alternative to use these approaches for the estimation of biological age^31–34^. However, in spite of their high accuracy, the way neural networks make predictions about the age of input samples is difficult to interpret, and their operation is somewhat analogous to a “black box” method, where we have no explanation regarding why some methylation profiles are estimated to be older or younger compared to others. The need for interpretable neural network-based methods has risen also in the broader field of computational biology, and a very promising advancement in this direction was achieved by Elmarakeby et al.^35^ by the introduction of a biologically informed deep learning tool for predicting the state of prostate cancer and evaluating molecular drivers of treatment resistance for therapeutic targeting. The suggested model used a huge collection of curated biological pathways to construct a pathway-aware multi-layered hierarchical deep learning network, thereby incorporating previously acquired biologically established hierarchical knowledge in a neural network language.

Inspired by this, here we propose a similar, biologically informed, explainable deep learning model for predicting the chronological age across multiple tissue types based on their methylation profiles. The structure of the neural network follows the hierarchy dictated by the biological pathways, in complete analogy with the tool presented by Elmarakeby et al.^35^. We compare the performance of the obtained method to that of elastic net regression in different use cases, including e.g., the data set by Gill et al.^36^ related to the rejuvenation of fibroblast cells. According to these studies, beside a slight gain in the prediction precision, the most important benefit of our approach is given by the versatile possibilities for comparing the importance of different CpGs, genes, biological pathways or entire pathway branches and layers in predicting the age across the human lifespan.

## Results

### Explainable deep-learning age prediction model

We created a deep learning prediction model named XAI-AGE (XAI stands for Explainable AI) that integrates previously identified biologically hierarchical information in a neural network model for predicting the biological age based on DNA methylation data. The training of the model relied on the available chronological age of the patients in the training set. The construction of this pathway-aware multilayered hierarchical network was based on 3007 manually curated biological pathways parsed from the Reactome Pathway Knowledgebase^37^. The individual’s molecular profile as DNA methylation beta values was entered into the XAI-AGE model as input and spread across a layer of nodes representing a set of genes through weighted links. This input layer can be extended in a modular way to incorporate multiple data modalities, such as gene expression, gene mutation status or other measurable features representable on the gene level.

**Figure 1:**
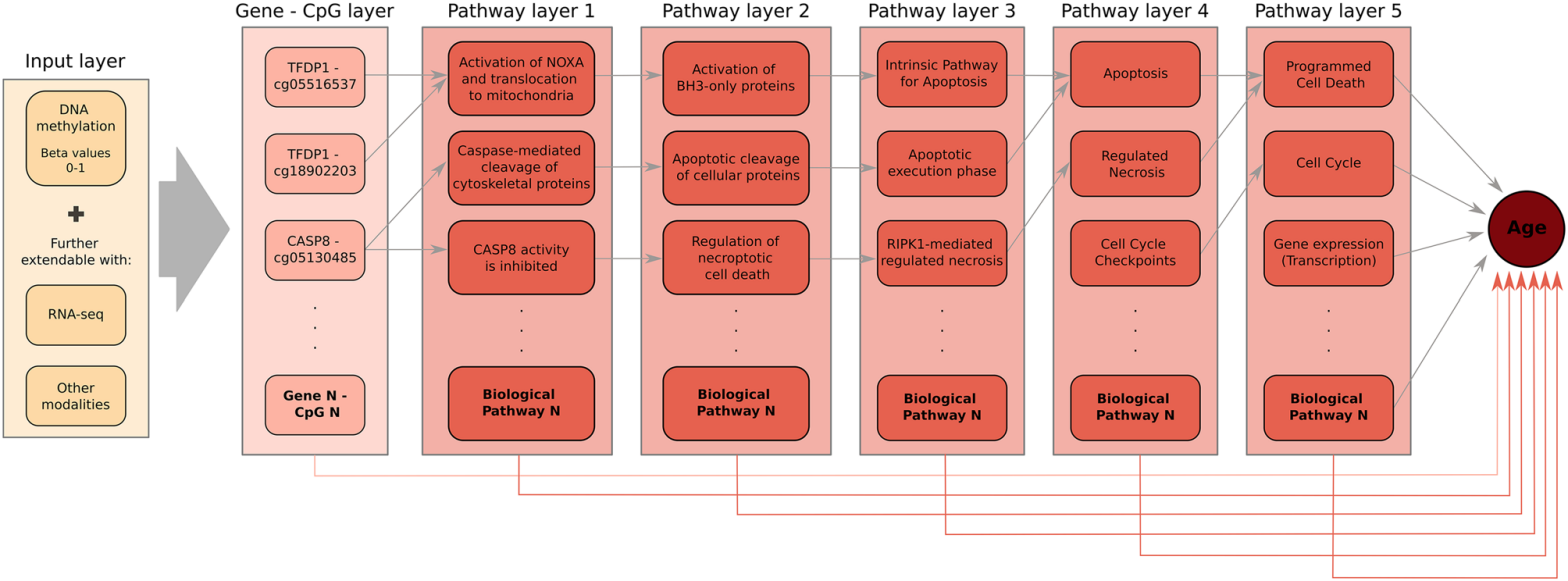
Schematic representation of the XAI-AGE model. The gene-CpG layer receives input data in the form of CpG methylation beta values. From here on, the information propagates in a restricted manner where nodes in the later layers are connected only if they are annotated jointly with the given node in the current layer according to the ReactomeDB. From left to right, the model’s abstraction becomes progressively more complex, as later levels in the neural network correspond to higher levels in the hierarchy defined by the ReactomeDB. The prediction of the chronological age for any given sample is given by the arithmetic mean of the outputs obtained for each individual layer. As indicated, the input layer can be easily extended by any new modalities which can be represented on the level of genes^35^.

Subsequent layers of the network encode a collection of routes with increasing degrees of abstraction, representing complicated biological activities. The layers closer to the input layer correspond to finer biological pathways and deeper layers represent the higher levels of the hierarchy in the Reactome Pathway Knowledgebase as illustrated in Fig.1. The connections between various layers are bound to follow known descendant-ascendant relations among encoded properties, genes, and pathways, making the network interpretable by design. The architecture of the model is shown in more details in Supplementary Table S2 in the Supplementary Material.

To determine the relative importance of particular genes, pathways and biological processes contributing to the model prediction, we examined each layer and used the DeepLIFT^38^ attribution approach to get the overall importance score of the neurons. Since the architecture is constrained by the underlying genes and biological processes, we can assume that the obtained importance scores can be used to test biological hypotheses across different subsets of the data. We note that the importance score is also a signed quantity, making it possible to infer trends in the dataset, however, the exact meaning of the direction is still not well understood. Hence, we included both positive and negative trends found during the analysis.

### Analysis of a pan-tissue data set

**Figure 2:**
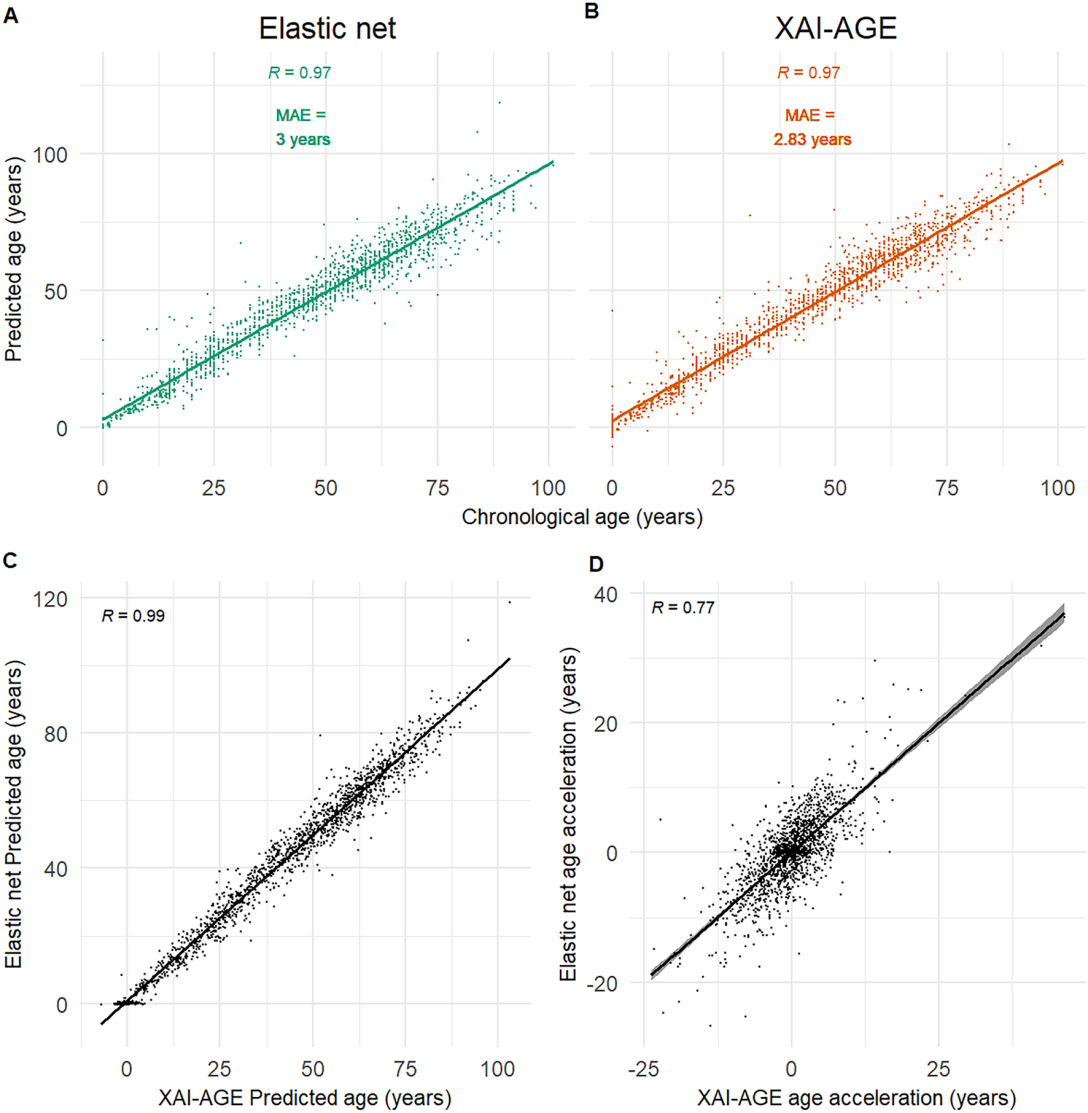
Predicted age as a function of the chronological age. We show the scatter plot of the age estimated based on the methylation data according to elastic net regression (**A**) and according to XAI-AGE (**B**). The predicted age and the age acceleration (defined by subtracting the chronological age from the predicted age) for the elastic net as a function of the same quantity according to the XAI-AGE model is also shown in (**C**,**D**). The Pearson correlation coefficients and the median absolute errors are indicated beside the plots, the number of samples were $n = 1619$.

The XAI-AGE model was trained and first tested on a pan-tissue data set (details are given in the Methods), and for comparison, an elastic net regression model similar to Horvath’s original regressor was also trained and evaluated on the same dataset. The performance of the two models was measured using the Pearson correlation coefficient and the median absolute error (MAE)^39^. As indicated in Fig. 2, we obtained 3 years MAE for the elastic net (Fig. 2A), and 2.83 years MAE for XAI-AGE (Fig. 2B) on the test set of the pan-tissue dataset, whereas the Pearson’s correlation coefficient was 0.97 for both models. Furthermore, the two models showed high correlation with each other as well when considering either the predicted age (Fig. 2C) or the age acceleration (Fig. 2D), defined as the difference between the predicted age and the chronological age. To further validate the XAI-AGE model’s performance, the results were replicated in a 5-fold cross-validation setting, where an artificial neural network, where all the neurons are connected between the layers (fully connected dense network) were trained as well and compared to XAI-AGE and the elastic net models (Supplementary Fig. S1). According to these tests, the MAE was significantly lower for XAI-AGE when compared to the elastic net model (Mann-Whitney U test, *p*-value = 0.028), while the dense fully connected neural network outperformed both models. However, it is important to note that the dense network contained more than 200 times more parameters. The neural network architecture for the fully connected dense model is shown on Supplementary Table S3.

The performance of the model was also examined by considering the various tissue types as displayed in Supplementary Fig. S2 in the Supplementary Material. By taking into consideration the varying amount of observations for the different tissue types, the results indicate that XAI-AGE provided the most accurate results for whole blood and blood PBMC tissue types, but performed poorly for blood cord, bone marrow, and esophagus.

Next, we investigated the explainable representations that XAI-AGE learnt from the pan-tissue cohort. Using the DeepLIFT attribution approach^38^, the feature importance scores were retrieved from each layer and neuron in the model. The top six characteristics that exhibited the greatest change between the beginning and the end of the timeline (from chronological age zero to the maximum of the cohort) were further classified based on whether they caused a positive or negative trend.

**Figure 3:**
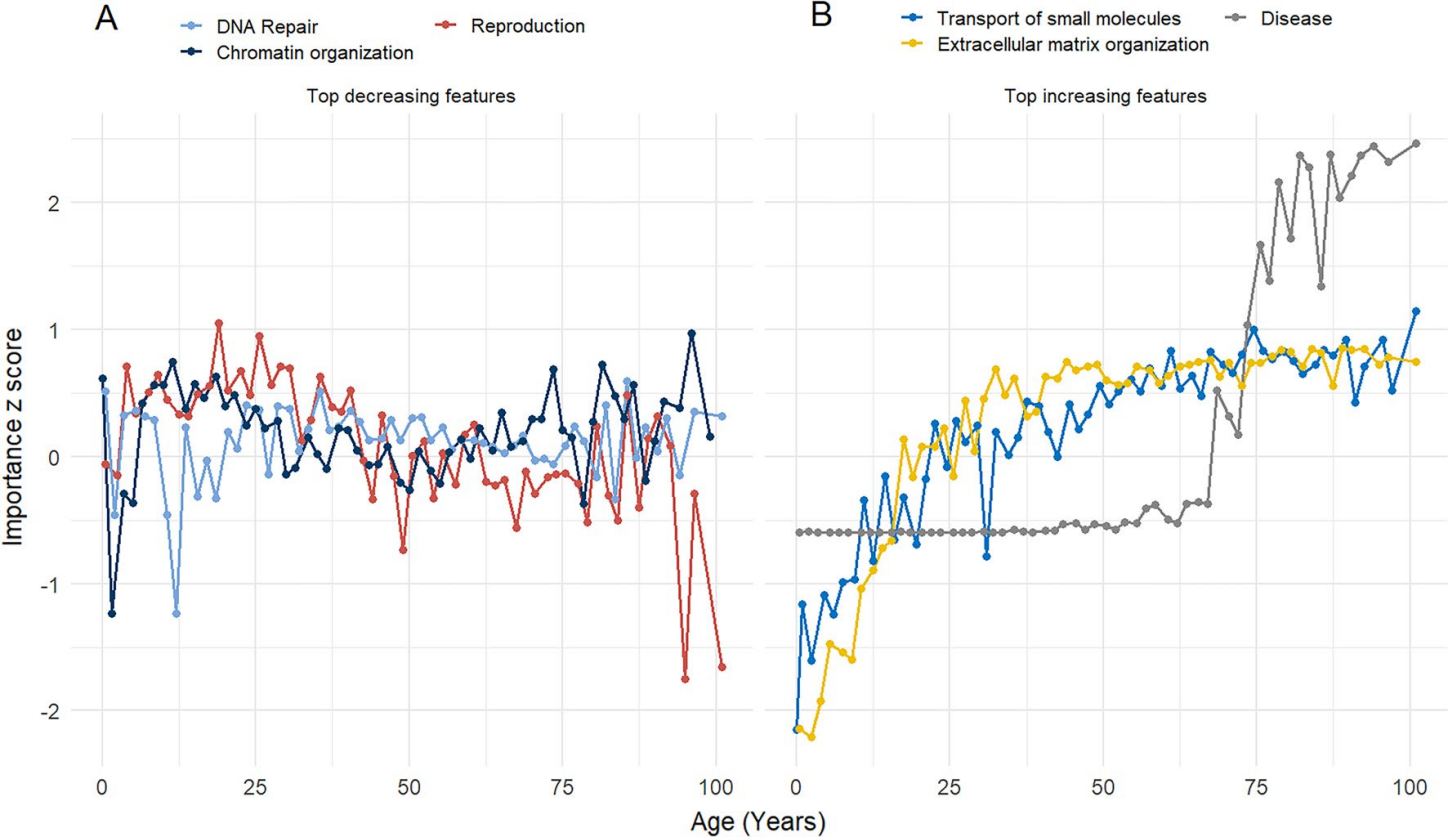
Standardized importance score as a function of chronological age. We show the *z*-score based on the distribution of the importance scores for the neurons (features) in the last layer of the network (corresponding to the top level in the hierarchy of the ReactomeDB) with the largest change in the *z*-score across the chronological age. The top 3 features where the trend in the *z*-score is negative are displayed in (**A**), whereas the top 3 features where the *z*-score is increasing with age are shown in (**B**).

In Fig. 3, we display the results for the last layer (corresponding to the top level in the hierarchy of the ReactomeDB), whereas similar plots for the other layers are presented in the Supplementary Material (Supplementary Figs. S3–S7).

From the features with a decreasing *z*-score over time, the top three features included the DNA Repair (R-HSA-73894), Chromatin organization (R-HSA-4839726) and the Reproduction (R-HSA-1474165) pathways. The top features where an increasing trend was observed in the *z*-score consisted of the Transport of small molecules (R-HSA-382551), Extracellular matrix organization (R-HSA-1474244) and a general pathway category called Disease (R-HSA-1643685). Interestingly, the latter exhibits a particular dynamics during the aging process, it remains constant until approximately the age of 70 then switches to a rapidly increasing tendency.

To demonstrate the advantages of XAI-AGE even further, a Plotly Dash graphical interface was built^40^, that renders Sankey plots similar to the one presented in Ref.^35^. This enables interactive navigation between the different layers of the network (each corresponding to a given level in the hierarchy of biological pathways according to the ReactomeDB), highlighting the features that contribute the most to the predictions (accessible at: https://k8plex-krft.vo.elte.hu/notebook/report/xgrp0j-sankeymethyl/).

**Figure 4:**
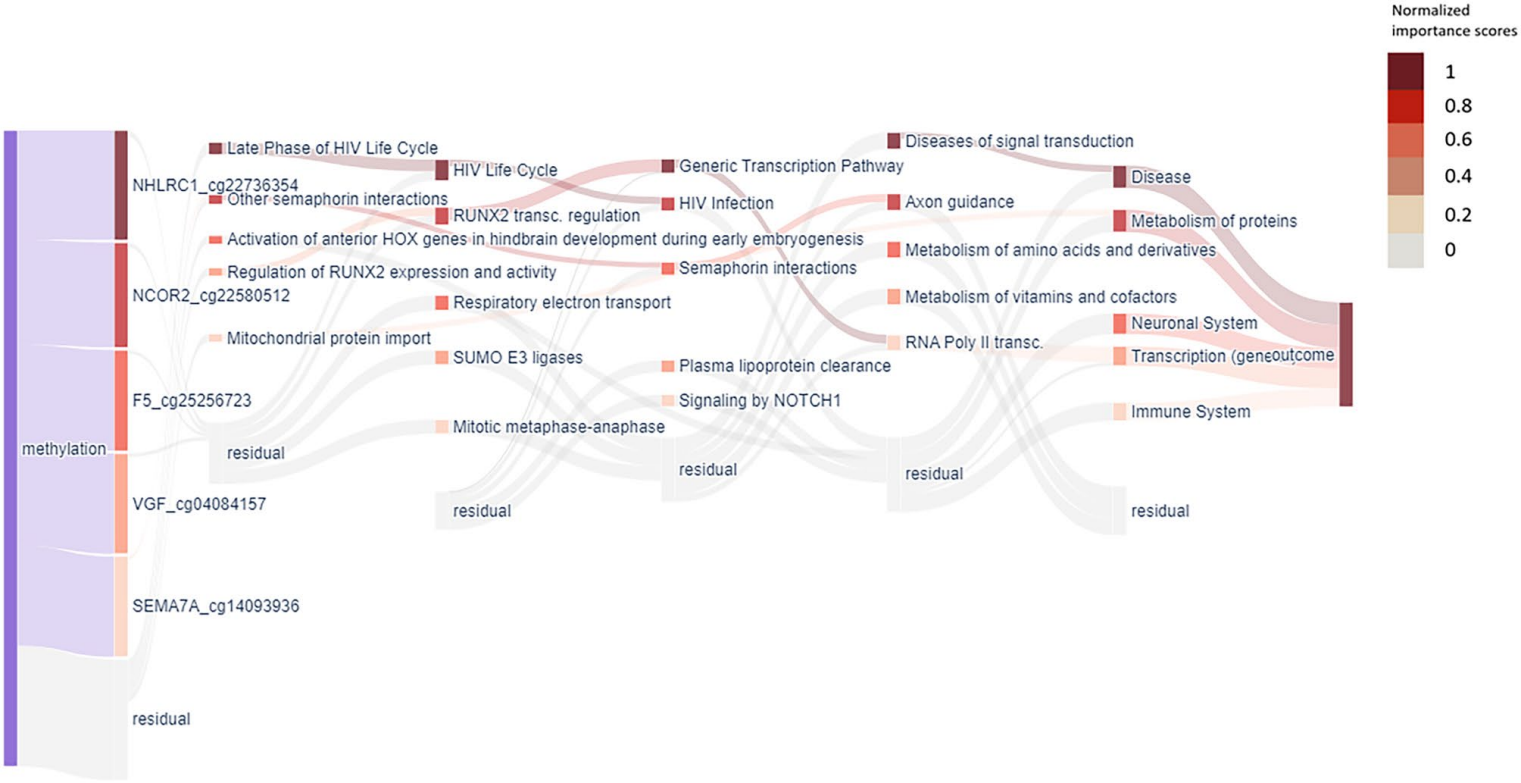
Sankey diagram of the top features calculated across all the samples in the pan-tissue dataset. The visualization of XAI-AGE layer structure shows the normalized relative importance score difference between the old (>65 years) and young samples for various nodes inside each layer. Darker colours indicate a larger difference between the importance scores for the two age groups at the given node. Only the top 5 nodes in each layer are displayed here, where the other nodes are indicated by the single semi-transparent nodes (named residual) at the bottom for each layer.

Since the links in this network indicate that the given pair of nodes are annotated to be related according to the ReactomeDB, one can track the flow of information between the layers, and infer the relevant sources that contributed to the prediction. As an illustration, in Fig. 4, we show the layer-wise standardized and ordered importance score for the samples in the pan-tissue dataset.

### Measuring the biological age during fibroblast reprogramming

**Figure 5:**
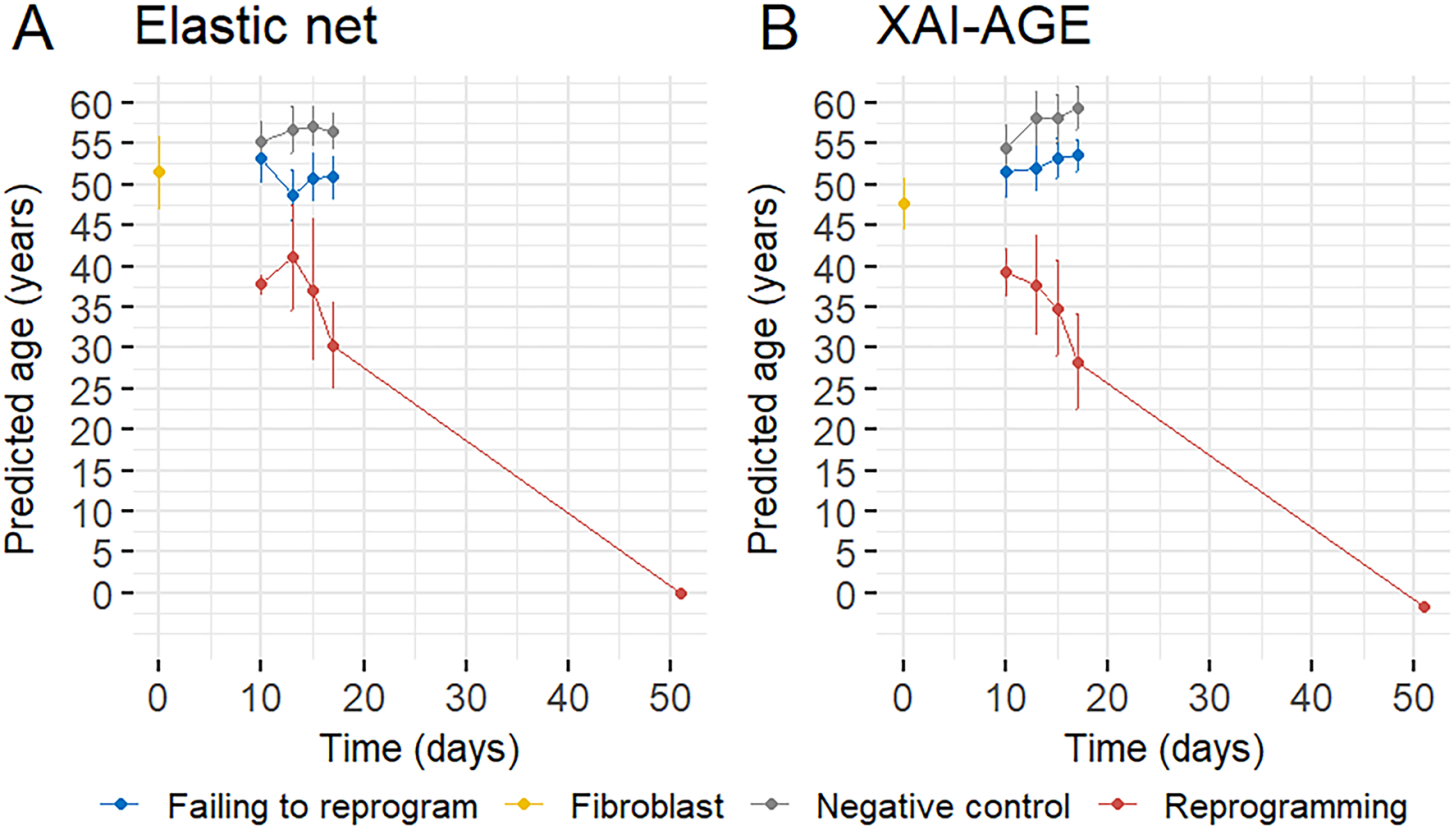
Comparing the biological age during the time-dependent differentiation process of human fibroblast cells from three donors. At time zero, one can observe undifferentiated fibroblast cells, and as time progresses, the biological age as determined by (**A**) elastic net and (**B**) XAI-AGE models are calculated. The cells can be further subdivided into categories that include fibroblasts utilized as negative controls, cells that failed to be transiently reprogrammed, and cells that were successfully transiently reprogrammed. The differentiation endpoint of induced pluripotent stem cells is also displayed on the final day of the measurement. The error bars represent the standard deviation across the three donors. A small positive trend can be also observed in the negative control group for the predicted age with slightly better correlation for XAI-AGE (R=0.38, p=0.0038) than for elastic net (R = 0.2, p = 0.23).

We also applied XAI-AGE to estimate the biological age of dermal fibroblast cells derived from middle age donors used in the reprogramming study by Gill et al.^36^. In this study, the cells were harvested for DNA methylation and RNA-sequencing in different time points during the reprogramming process. In the present study we used the methylation data for calculating the biological age predicted by XAI-AGE for both the treated and the (non-treated) control cells. According to Fig. 5, our age estimation framework gave results similar to that obtained by the Horvath clock-like elastic net. Both epigenetic clocks precisely predicted the biological age of the cells in the negative control and failed to reprogram group, as well as a significant drop for the transiently reprogrammed cells. However, according to the original study by Gill et al.^36^, the methylation levels go significantly down across all gene groups for these cells, which could provide a simple explanation for this effect. As anticipated, the predicted biological age of the iPSC cells was close to zero. Interestingly, the predicted biological age of the negative control cells shows a positive trend in time, consistent with the recent findings by Levine et al.^41^.

**Figure 6:**
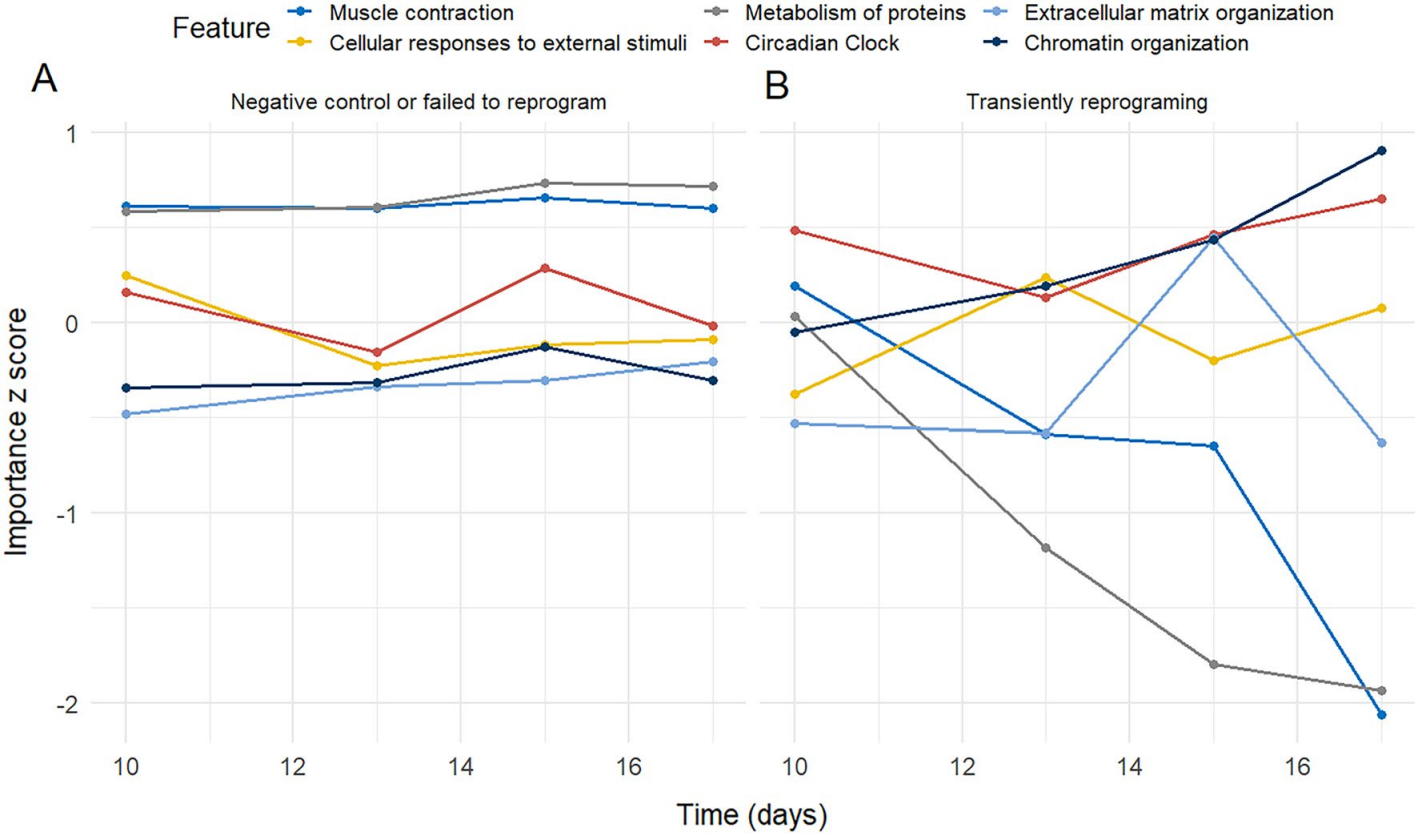
Time dependence of the standardised importance score values in the human fibroblast experiment. We show the six scores from the last layer of the network where the largest deviation can be observed between the negative control or the failed to reprogram group (**A**) and the transiently reprogrammed group (**B**).

Similarly to the analysis of the pan-tissue cohort, we calculated the importance scores for both the individual neurons and all the layers. This allowed the study of the importance in the age prediction of the different features and biological pathways during the reprogramming process. The results for the last layer in the neural network (highest level in the biological pathway hierarchy) are displayed in Fig. 6, showing the top six features according to the magnitude of the change over time for the negative controls and unsuccessfully reprogrammed cells (Fig. 6A), as well as for the transiently reprogrammed cells (Fig. 6B). The time-dependent dynamics of the importance scores shows interesting differences between the negative controls and the reprogrammed cells, e.g., the Metabolism of proteins (R-HSA-392499) and Muscle contraction (R-HSA-397014) significantly changed in the negative direction in the transiently reprogramming group, while the Chromatin organization (R-HSA-4839726) and Circadian clock (R-HSA-400253) increased. The extracted importance scores from the other layers of the model can be seen on Supplementary Figs. S8–S12.

### Biological age in umbilical cord plasma transfusion

As a further application of XAI-AGE, we also analysed a recently published dataset by Clement et al.^42^, related to umbilical cord plasma transfusion. Heterochronic parabiosis studies have shown favorable benefits in aged animals getting youthful blood across a variety of tissues^43^. The study presented in Ref.^42^, examined whether infusion of plasma or plasma-derived factors from young donors could be used to mitigate human age-related conditions by administering human umbilical cord plasma concentrate to elderly patients (n = 18, mean age = 74) and monitoring epigenetic age-related measures for a period of 10 weeks. The authors have shown that the treatment lowered DNA methylation-based GrimAge measure by an average of 0.82 years, indicating a decrease in the risk of morbidity and mortality. However, other epigenetic clocks that estimate chronological age did not detect a significant age-reversal effect.

In the present work, using this data, we first estimated the chronological age of the individuals using XAI-AGE. The comparison between the predicted age and the chronological age stratified by the pre-treatment and post-treatment samples is shown in the Supplementary Material in Supplementary Fig. S13, indicating a high correlation between the two variables. Next, we compared the age acceleration (corresponding to the difference between the estimated value and the actual chronological age) predicted by XAI-AGE between the two groups of samples derived from the same individuals, similarly as was described in^42^. A paired t-test was performed and reported no significant changes.

Furthermore, the importance score for each feature in each layer was extracted and compared between the pre-treatment and post-treatment groups. In Supplementary Figs. S14–S19, the six top features from the last layer according to the magnitude of the difference between the two groups are shown, of which three correspond to the top features where this difference is positive, and the other three are the top features where the difference is negative. Our results indicate that the Cell-cycle (R-HSA-1640170), Cell-Cell communication (R-HSA-1500931) and the Reproduction (R-HSA-1474165) pathways were more important in the post-treatment samples, while the Circadian clock (R-HSA-400253), Mitophagy (R-HSA-5205647) and the Vesicle-mediated transport (R-HSA-5653656) pathways were more important in the pre-treatment group. Overall, the XAI-AGE results are less informative for this data set that may indicate that either the input data is not robust enough or may indicate weak points of XAI-AGE.

More extended data analyses of the results and the comparison of the importance scores from the other layers of the network are described in the Supplementary Materials.

## Discussion

In this paper, we present an accurate and explainable neural network architecture allowing not only the estimation of age based on DNA methylation data with high precision but also the easy interpretation of results that are comparable across tissues, age groups, and differentiation processes in the case of cell lines. The resulting model can be used to generate hypotheses and visualize the underlying mechanisms connected to aging. We have demonstrated this feature of the model by examining the importance scores of the individual neurons in predicting the age when the neural network was trained on different datasets. In this aspect, probably the most noteworthy result was obtained for the pan-tissue dataset, where the standardised importance score for the Disease pathway (corresponding to a neuron in the last layer of the neural network) displayed a particular behaviour when plotted as a function of age, showing a roughly constant flat curve that is replaced by a rapidly increasing function at the age of 70.

The second important observation is related to the DNA Repair pathway, which demonstrated a decreasing tendency in the pan-tissue cohort when the importance *z*-score was visualized as a function of age (Fig. 3A). The DNA repair pathway is part of the DNA damage response system that is responsible for the maintenance of genome integrity. Living organisms are constantly exposed to exogenous and endogenous DNA damage. Unrepaired or faulty repair of DNA damage leads to the accumulation of somatic mutations as an organism ages, making genome instability a hallmark of aging^1^. The importance of DNA repair mechanisms to counteract the time- and exposure-dependent accumulation of DNA damage is highlighted by the fact that inherited mutations in genes that are involved in these pathways underlie several segmental premature ageing-like syndromes in humans^44^. Our result is in agreement with accumulating evidence suggesting that the integrity and maintenance of the genome are strongly associated with aging^45,46^. The Chromatin organization pathway was also selected as one of the top decreasing features in the last layer of the network based on the change in the importance *z*-score across the chronological age of the individuals (Fig. 3A). The Chromatin organization pathway includes chromatin modifying enzymes involved in processes that result in the specification, formation or maintenance of the physical structure of eukaryotic chromatin. The identification of this pathway as one of the top features in the XAI-AGE network is coherent with the well-established fact that epigenetic changes affecting DNA methylation patterns, histone modifications and chromatin remodeling are the hallmarks of ageing^1^.

Biological pathways that demonstrated the largest difference between the importance scores of the old (> 65 years) and young samples are shown in Fig. 4. In the third layer, one of the top 5 nodes is the Mitotic metaphase and anaphase pathway that regulate the proper segregation of chromosomes into daughter cells. Recently, several epigenetic mitotic clocks were developed, such as epiTOC^47^, epiTOC2^48^ and solo-WCGW^49^. epiTOC and epiTOC2 rely on CpG sites in CpG-rich regions that are marked by the polycomb repressive complex 2 (PRC2), which are generally unmethylated across numerous different fetal tissue types, to calculate the rate of stem cell division^47,48^. On the other hand, solo-WCGW focuses on DNA methylation loss at partially methylated domains (PMDs) that showed increased hypomethylation with age and appeared to track the accumulation of cell divisions^49^. It seems that the identification of the Mitotic metaphase and anaphase pathway as significantly different between old and young individuals by the XAI-AGE model captures a different association between mitotic processes and ageing than the previously described epigenetic mitotic clocks since we did not identify overlapping genes between the Mitotic metaphase and anaphase pathway and the described epigenetic mitotic models. However, a detailed analysis of these interesting findings is to be explored in further studies.

We calculated the standardised importance scores from the last layer of the XAI-AGE model using the data from a fibroblast rejuvenation experiment^36^. The largest difference between the negative control or failed to reprogram group and the transiently reprogrammed group were observed in six biological pathways (Fig. 6). Among these pathways are the Extracellular matrix organization and the Muscle contraction pathways that likely to reflect the observations made by Gill and colleagues that the reprogrammed fibroblasts produced youthful levels of collagen proteins, and showed partial functional rejuvenation of their migration speed^36^. Interestingly, the Circadian clock pathway and several known associated pathways, such as the Cellular response to external stimuli, Chromatin organization and Metabolism of proteins, were also identified as important by the XAI-AGE model in the fibroblast reprogramming process during which the DNA methylation age measured by the multi-tissue epigenetic clock was significantly decreased^36^. The circadian clock is an endogenous, biological timing mechanism that responds to several external stimuli to maintain the synchronization of internal biological processes among themselves and with exogenous environmental cycles^50^. The core clock genes, including *CLOCK1*, *BMAL1*, *PER* and *CRY* genes, are rhythmically expressed and form a negative feedback loop that drives circadian oscillations.

The underlying transcription-translation feedback system of the circadian clock regulates the expression of clock-controlled genes that are involved in various processes, e.g., metabolism and chromatin remodelling^51^. A growing body of evidence suggests a link between the disruption of the circadian rhythms and ageing. Studies have shown that disturbances in the circadian clock and sleep homeostasis are linked to increased incidence of a variety of age-related health problems, such as neurodegenerative diseases, metabolic disorders, cardiovascular disease, obesity and cancer^52–55^. Furthermore, the transcription factor *BMAL1*, which is the co-activator of the circadian clock, exhibited decreased regulatory activity with age independently from cell-type and tissue-type^56^.

According to the chrono-epigenetic theory, circadian oscillations of cytosine modification at specific CpG sites are robust in young individuals but diminish with age, potentially as a result of changed activity of ten-eleven translocation (TET) and DNA methyltransferase (DNMT) maintenance enzymes. Age-related changes in amplitudes of the oscillations precede linear DNA methylation changes and might predict age-dependent linear outcomes^57^. Our results suggest that the synchronization of oscillatory rhythms of internal biological processes is associated not only with ageing but also with rejuvenation of human cells by maturation phase transient reprogramming.

Additional advantages of the model include the modular construction of the underlying neural network: the input layers can be modified to incorporate additional modalities, allowing the integration of multiomics data, as demonstrated in an analogous example by Gill et al.^36^. In the case of age prediction, the logical next step would be to include RNA-seq data alongside DNA methylation values in the model. This can be easily accomplished by vertically increasing the input layer in the model and making the new data modalities representable at the level of genes. This modularity applies to the deeper layers in the model (corresponding to higher levels in the pathway hierarchy according to the ReactomeDB) as well. The core structure in the Reactome Pathway can be freely altered, enlarged, or replaced by another database. Along this line, the incorporation of the so-called Hallmarks of Aging^1^ into the interaction network to make it more aging-specific is an intriguing study topic for the future.

There are also some limitations to our analysis, e.g., compared to other deep learning based models like DeepMAge optimised solely for prediction accuracy, XAI-AGE performs worse by around half a year MAE^34,58^. Regarding our training data, there are substantial class imbalances of tissues and age groups, and batch effects from the various data sources that may be included can potentially bias the results. A curation bias can also alter the results of the Reactome Pathway Database, which is another issue. For instance, the HIV pathway was over-represented in our data, which may play little role in predicting the biological age because the same critical age-related genes are present in several pathways and the neural network amplifies the value of these neurons for better prediction. Pre-training the neural network on the CpG data level (for instance, by changing the architecture to an Autoencoder) and then fine-tuning it to predict the biological age is a potential solution to this issue.

Clarifying the causal relationship between the many CpG-s, genes, and biological processes associated with aging would be a future goal for biologically informed deep learning approaches. The primary advantage of XAI-AGE over other epigenetic clocks is the direct comparison and inference of relationships between more abstract data layers than using raw input data alone. Further supplementation of the model with additional biological data modalities, such as incorporating RNA-seq at the gene level or evaluating the data as a time series, as we demonstrated with the fibroblast reprogramming dataset, could facilitate the future discovery of causal relationships. Using XAI-AGE could assist by analyzing computationally its interpretable network, or by domain experts using the Sankey diagram interactive visualization.

## Methods and data

### Applied data sources in this study

All data used in the study is publicly available. The complete list of the data sources are shown in Supplementary Table S1. All methods were carried out in accordance with relevant guidelines and regulations.

### Training the model on the pan-tissue data set

We trained and tested XAI-AGE with a set of 6547 patient samples across 54 cohorts and multiple tissues (Supplementary Table S1), divided into 75% training, 25% testing, to predict the chronological age based on the DNA methylome of the individuals. This estimation was later used to also infer the biological age, defined by the chronological age prediction of the model.

### Fibroblast cell reprogramming data

In the study by Gill et al.^36^ the cells were harvested for DNA methylation and RNA-sequencing in different time points during the reprogramming process. Altogether 96 cells were analysed during the study from three different individuals which can be further subdivided into the categories of cells that were measured prior to the reprograming phase (fibroblasts), negative controls that received mock treatment, cells that failed to reprogram, and cells that transiently reprogrammed successfully. Cells that had been fully reprogrammed (iPSC) and were sampled on the final day were also measured.

### Umbilical cord plasma transfusion data

The dataset contains 36 whole-blood samples collected at the beginning and at the end of the 10-week experiment period. In the present study, we used the already trained XAI-AGE model to estimate the biological age and biological age acceleration for each sample, and the results were compared between the pre-treatment and post-treatment groups.

### Supplementary Information

Supplementary Information 1.

Supplementary Information 2.

Supplementary Information 3.

Supplementary Information 4.

Supplementary Information 5.

Supplementary Information 6.

## Data Availability

All data used in the study were downloaded from the Gene Expression Omnibus (https://www.ncbi.nlm.nih.gov/geo/) and from the The Cancer Genome Atlas data portal (https://portal.gdc.cancer.gov/) databases. The corresponding dataset ID-s are listed in the supplementary information file. Any data not public can be requested.

## Code availability

The code for running inferences with the XAI-AGE model can be accessed at: https://github.com/Paureel/XAI-AGE.
