# Supplementary material for: Biologically informed deep learning for explainable epigenetic clocks
Source: Sci Rep. 2024 Jan 15;14:1306. doi: 10.1038/s41598-023-50495-5 (PMC10789766; doi:10.1038/s41598-023-50495-5)
Supplement: Supplementary file 1 — Supplementary Information 1. [file 41598_2023_50495_MOESM1_ESM.pdf]

Biologically informed deep learning for explainable epigenetic clocks - Supplementary material

Aurel Prosz
Danish Cancer Society Research Center, Copenhagen, Denmark

Orsolya Pipek
Dept. of Physics of Complex Systems, ELTE Eötvös University, Budapest, Hungary

Judit Börcsök
Danish Cancer Society Research Center, Copenhagen, Denmark,
Biotech Research
Innovation Centre (BRIC), University of Copenhagen, Copenhagen, Denmark

Gergely Palla
Department of Biological Physics, ELTE Eötvös Loránd University, Budapest, Hungary,
Health Services Management Training Centre, Semmelweis University, Budapest, Hungary

Zoltan Szallasi
Danish Cancer Society Research Center, Copenhagen, Denmark

Sandor Spisak
Institute of Enzymology, HUN-REN Research Centre for Natural Sciences, Budapest, Hungary

Istvan Csabai
Dept. of Physics of Complex Systems, ELTE Eötvös University, Budapest, Hungary

* = Correspondence should be addressed: spisak.sandor@ttk.hu

10 25, 2023

S1 Supplementary methods, tables and figures

In Supplementary Table 1, a comprehensive enumeration of the datasets employed for the purposes of training and evaluating the model is provided. This tabulation offers a detailed overview of the specific datasets utilized in the model's development and subsequent testing phase, allowing for an understanding of the data sources that contributed to the model's performance.

Supplementary Tables 2 and 3 present the neural network architecture for the XAI-AGE model and the fully-connected dense neural network model, respectively. These tables elucidate the structural components of each model, providing a clear depiction of their respective configurations, input and output parameter counts of each layer.

Supplementary table S1: List of the datasets used for the training and testing of the model.

Study ID	Tissue	Sample count
TCGA-PCPG	Adrenal Gland	3
TCGA-CHOL	Bile Duct	9
TCGA-BLCA	Bladder	21
GSE27317	Blood Cord	168
GSE34257	Blood Cord	84
GSE36642	Blood Cord	53
GSE36812	Blood Cord	48
GSE27097	Blood PBMC	386
GSE30870	Blood PBMC	38
GSE32149	Blood PBMC	46
GSE36064	Blood PBMC	78
GSE20067	Blood WB	188
GSE20236	Blood WB	93
GSE40279	Blood WB	656
GSE41037	Blood WB	716
GSE41169	Blood WB	95
GSE42861	Blood WB	689
GSE19711	Blood WB	534
TCGA-GBM	Brain	1
GSE38873	Brain CRBLM	168
GSE15745	Brain CRBLM, Brain FCTX, Brain PONS, Brain TCTX	498
GSE41826	BrainVariousCells	145
TCGA-BRCA	Breast	122
GSE32393	Breast NL	23
GSE25892	Buccal	109
GSE42700	Buccal	53
TCGA-CESC	Cervix	3
GSE32146	Colon	24
TCGA-COAD	Colorectal	75
TCGA-READ	Colorectal	12
GSE22595	Dermal fibroblast	14
TCGA-ESCA	Esophagus	16
GSE38291	Fat Adip, Muscle	30
TCGA-HNSC	Head and Neck	50
TCGA-KIRC	Kidney	356
TCGA-KIRP	Kidney	50
TCGA-LIHC	Liver	49
TCGA-LUAD	Lung	54
TCGA-LUSC	Lung	69
GSE17448	MSC (bonemarrow)	16
TCGA-OV	Ovary	4
TCGA-PAAD	Pancreas	10
GSE36642	Placenta	28
GSE44667	Placenta	40
TCGA-PRAD	Prostate	50
GSE28746	Saliva	69
GSE34035	Saliva	183
TCGA-SKCM	Skin	2
TCGA-SARC	Soft Tissue	4
TCGA-STAD	Stomach	27
TCGA-THYM	Thymus	2
TCGA-THCA	Thyroid	56
GSE30758	Uterine Cervix	151
TCGA-UCEC	Uterus	35
GSE36166	various tissues	44
GSE165180	fibroblasts	96
GSE210245	whole blood	36

Supplementary table S2: The artificial neural network architecture of the XAI-AGE model. Total parameters: 101645

Layer name	Type	Output shape	Number of parameters	Connected to
inputs	InputLayer	15591	0	
h0	Diagonal	15591	31182	inputs
dropout_0	Dropout	15591	0	h0
h1	Dense	1387	47046	dropout_0
dropout_1	Dropout	1387	0	h1
h2	Dense	1066	2462	dropout_1
dropout_2	Dropout	1066	0	h2
h3	Dense	447	1517	dropout_2
dropout_3	Dropout	447	0	h3
h4	Dense	147	594	dropout_3
dropout_4	Dropout	147	0	h4
h5	Dense	26	174	dropout_4
o_linear1	Dense	1	15592	h0
o_linear2	Dense	1	1388	h1
o_linear3	Dense	1	1067	h2
o_linear4	Dense	1	448	h3
o_linear5	Dense	1	148	h4
o_linear6	Dense	1	27	h5
o1	Activation	1	0	o_linear1
o2	Activation	1	0	o_linear2
o3	Activation	1	0	o_linear3
o4	Activation	1	0	o_linear4
o5	Activation	1	0	o_linear5
o6	Activation	1	0	o_linear6

Supplementary table S3: The artificial neural network architecture of the fully connected dense model. Total parameters: 23702217

Layer name	Type	Output shape	Number of parameters	Connected to
inputs	InputLayer	15591	0	
h0	Diagonal	15591	31182	inputs
dropout_0	Dropout	15591	0	h0
h1	Dense	1387	21626104	dropout_0
dropout_1	Dropout	1387	0	h1
h2	Dense	1066	1479608	dropout_1
dropout_2	Dropout	1066	0	h2
h3	Dense	447	476949	dropout_2
dropout_3	Dropout	447	0	h3
h4	Dense	147	65856	dropout_3
dropout_4	Dropout	147	0	h4
h5	Dense	26	3848	dropout_4
o_linear1	Dense	1	15592	h0
o_linear2	Dense	1	1388	h1
o_linear3	Dense	1	1067	h2
o_linear4	Dense	1	448	h3
o_linear5	Dense	1	148	h4
o_linear6	Dense	1	27	h5
o1	Activation	1	0	o_linear1
o2	Activation	1	0	o_linear2
o3	Activation	1	0	o_linear3
o4	Activation	1	0	o_linear4
o5	Activation	1	0	o_linear5
o6	Activation	1	0	o_linear6

S1.0.1 XAI-AGE design and training

Similarly as first introduced by the P-NET architecture [1], the first layer of XAI-AGE is connected to the second layer by a set of one-to-one connections, and each node in the second layer is connected to exactly as many nodes of the input layer as many CpG-s were annotated for a given gene using the *minfi* R package [2] and each input-node represents a different beta value for a specific CpG site. CpG sites which were not mapped to any genes were discarded. The output of each layer is determined as

$$y = f[(M * W)^T X]$$

, where f represents the activation function, M represents the mask matrix derived from the Reactome DB, W represents the weights matrix, X represents the input matrix, b represents the bias vector, and $*$ represents the Hadamard product. The f activation function is chosed to be the *tanh* function to keep the activation values in the $[-1, 1]$ range, and the outcome nodes have a linear activation.

To allow each hidden layer to be useful on its own, a predictive layer with sigmoid activation was added after each hidden layer, and the weighted average was determined for the final prediction. The model was trained to minimize the mean squared error between the chronological age and the predicted age by the neural network.

The learning rate was initialized at 0.001 and actively decreased after every 250 epochs to facilitate smooth convergence, and the Adam optimizer was used to train the model.

During the inference, the DeepLIFT method was chosen to rank the features in all the layers of the neural network. DeepLIFT is a backpropagation-based attribution method that assigns a significance value at the sample level to each feature [3]. The objective of this method is to award scores to each node in each layer, which is realized by calculating an importance score for each node on based on the difference in between the target activation t and a reference value t_0 such that the difference equals the aggregate of the calculated scores for all nodes. As suggested by Elmarakeby et al. [1], we modified the DeepLIFT scores using a graph-informed function that takes each node's connectedness into account. To obtain an aggregated importance score on the samples to compare between tissues and age groups, the arithmetic average was calculated.

For the training of the elastic net model which is served as a baseline and has a similar methodology as the Horvath clock, we used the *ElasticNet* function from the glmnet package [4], with default parameters. The algorithm returned 683 non-zero weights.

We also trained a fully-connected dense model with the same number of nodes as the sparse XAI-AGE model, while still keeping the first layer sparse, since a dense network having an equal quantity of nodes would possess over 200 million weights, where the weights would be concentrated mostly in the first layer. This newly trained, fully-connected dense model with sparse first layer contained more than 200 times more parameters, since all the nodes are connected to all the others between two consecutive layers.

S1.0.2 Data pre-processing

The data for training and evaluating the XAI-AGE model were obtained from public repositories from the Gene Expression Omnibus [5] database and from the The Cancer Genome Atlas Program official repository [6]. For both models the beta values were used as the input, and the chronological age from the metadata as the target variable, which was transformed by the same logarithmic function as described in [7]. For the pan-tissue dataset, we splitted each cohort to 75% training and 25% testing set, and aggregated the two together to obtain the final training and test set. For the fibroblast transient reprograming data we simply used the trained models on the pan-tissue dataset, and calculated the predicted age for each cells.
As a metric to evaluate the test data, the Pearson correlation coefficient and the median absolute error (MAE) were used, where the MAE is defined as for n samples:

$$MAE(y, \hat{y}) = median\left(|y_1 - \hat{y}_1|, ..., |y_n - \hat{y}_n|\right)$$

S1.0.3 Obtaining the importance scores for interpreting the network

The importance scores were calculated on the sample level for all test samples in the pan-tissue dataset, for all cells in the cell reprogramming cohort and for all cases in the umbilical cord plasma concentrate analysis. In the first set, the importance scores for all samples independently from each other were calculated. Next, the samples were ordered by their respective chronological age, and the top 3 largest positively, and top 3 largest negatively changing features given their z-score transformed importance scores were extracted from each layer of the network. This was done by calculating the difference of the standardized importance scores at the beginning of the time series and at the end, and the features having the largest differences were selected. These selected features were then aggregated by their median

if they assigned to the same chronological age and then plotted and analyzed in the function of the chronological age. For the reprogramming dataset, the cells were grouped together either by the days since the reprogramming began, and if they were successfully reprogrammed, failed, or belong to the control group. The examination of the important features were done similarly as in the first cohort, but this time the days since the initiation of the reprogramming was considered instead of the chronological age, and the comparison was done between the grouping as described above, aggregating the samples by median for the three individuals.
Lastly, the same method was applied for the umbilical cord plasma concentrate dataset, but instead of time, only the pre and post treatment groups were compared to each other and the top three features were analyzed given their importance score, in both positive and negative trend.

S1.0.4 Statistical analysis and R packages

The XAI-AGE framework was developed using Python 2.8 and Tensorflow. Using the Plotly python package, the Sankey-plots were rendered. The ggplot2 and ggpubr programs were utilized for plotting and R was used for statistical analysis to produce correlation coefficients, MAE, and t-tests.

S1.1 Supplementary figures

In Supplementary Figure 1, the outcomes of five-fold cross-validation experiments are presented with the intent of approximating the error rate and the efficacy of three distinct models: the XAI-AGE model, the elastic net model, and the fully connected dense neural network model. This estimation process allows for a comprehensive evaluation of each model's performance.

Supplementary Figure 2 displays a scatter plot that demonstrates the estimated biological age as determined by the XAI age model. This visualization encompasses various tissues found within the pan-tissue dataset, elucidating the distribution of estimated biological ages across these distinct tissue types.

Supplementary Figures 3 through 7 illustrate the standardized importance scores over the chronological age within the test set of the pan-tissue dataset. Each figure showcases only one layer of the network. On the left side, the top three decreasing features are displayed, whereas the right side presents the top three increasing features, spanning from the inception of the dataset to the maximum age contained therein.

Turning to Supplementary Figures 8 through 12, these images depict the standardized importance scores throughout the days measured, commencing from the initiation of the fibroblast reprogramming experiment. Each figure highlights one layer of the network at a time, while simultaneously exhibiting the six features that exhibit the most considerable differences.

In Supplementary Figure 13, the age acceleration predictions generated by the XAI-AGE model are portrayed within the context of the umbilical cord plasma transfusion dataset. This representation allows for an assessment of the model's ability to predict age acceleration based on the available data.

Finally, Supplementary Figures 14 through 19 display the six features that contribute either the largest disparities between post-treatment and pre-treatment groups or the most significant differences between pre-treatment and post-treatment within the umbilical cord plasma transfusion dataset. This visualization enables an examination of the impact of these specific features on the overall results.

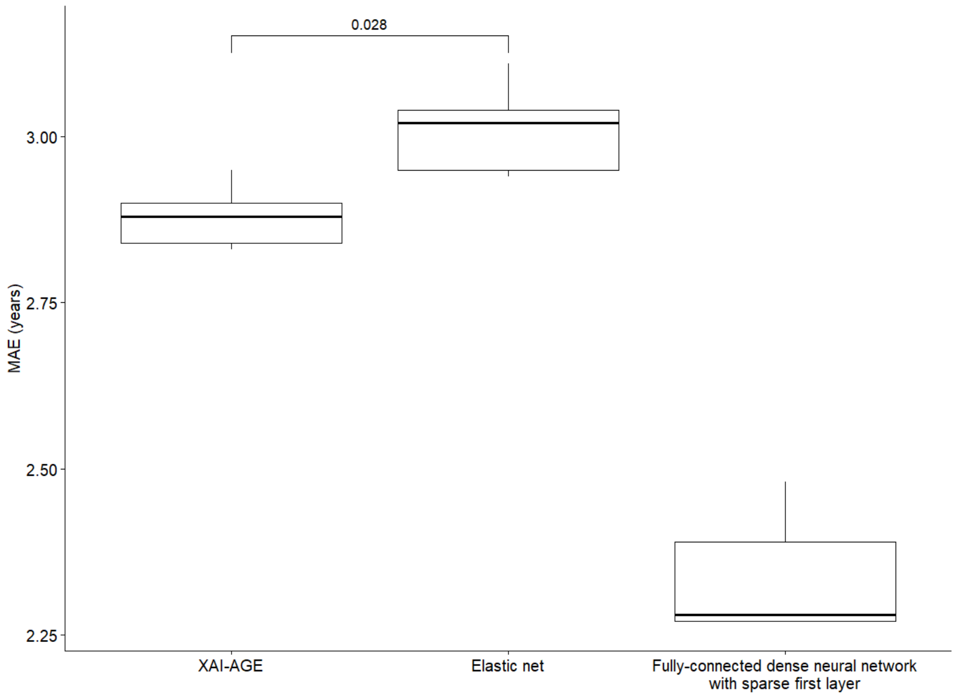

Supplementary figure S1: Results of five-fold cross-validation experiments to estimate the error rate and performance of the models. Comparison of Mean Absolute Error (MAE) between XAI-AGE, Elastic Net models and the fully connected dense neural network with sparse first layer models. The MAE for XAI-AGE was significantly lower than that of the Elastic Net model (Mann-Whitney U test, p-value = 0.028). The dense fully connected neural network architecture demonstrated superior performance compared to both models.

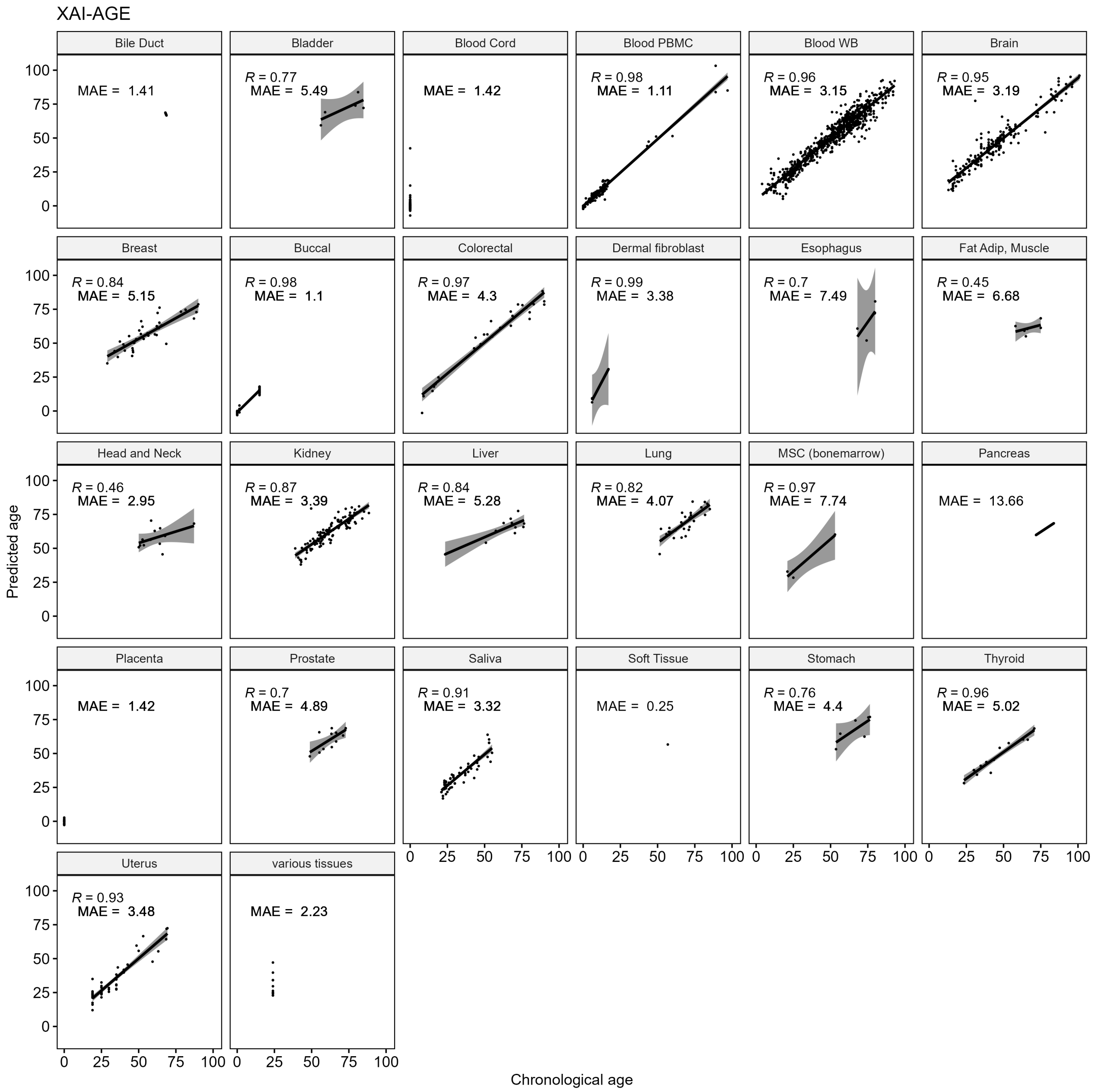

Supplementary figure S2: **Predicted age for different tissues.** The scatter plot of the estimated biological age obtained using XAI age is shown for the different tissues appearing in the pan-tissue dataset in separate panels, where the tissue type is indicated in the panel title.

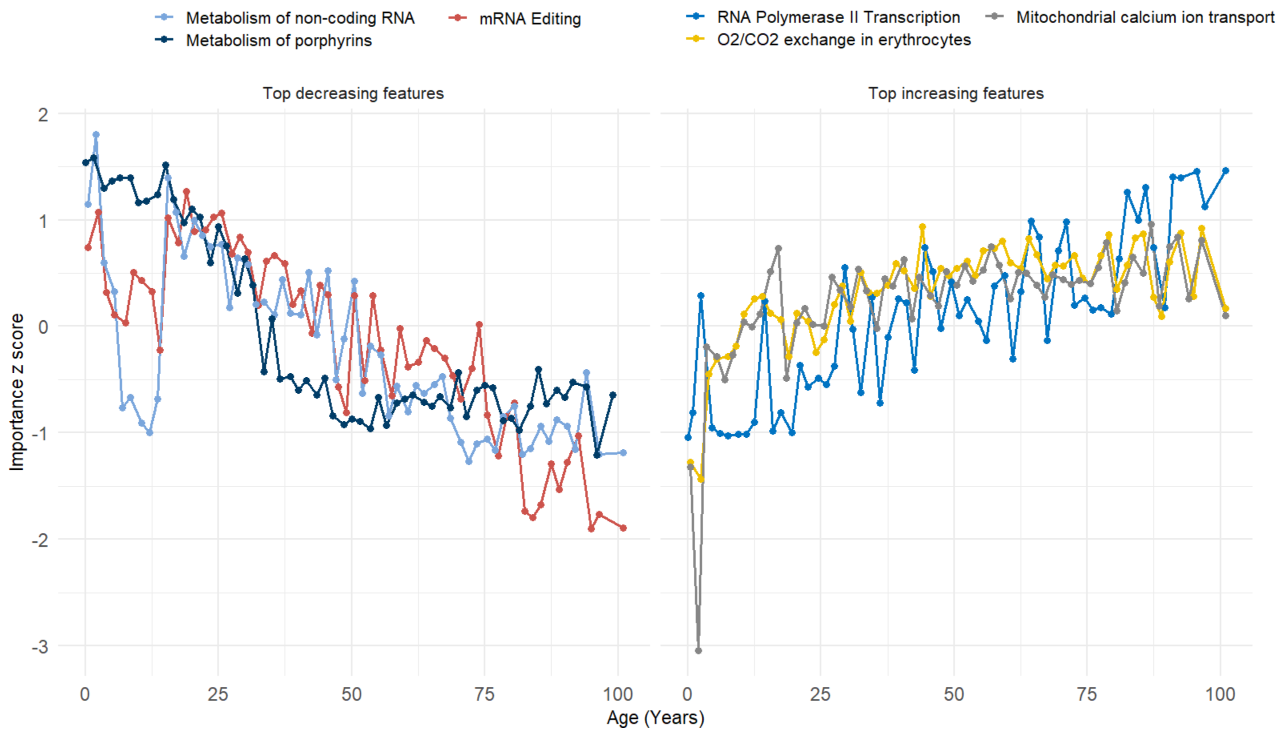

Supplementary figure S3: Visualizing the standardized importance scores across the chronological age on the test set of the pan-tissue dataset, showing only the fifth layer of the network. On the left the top 3 decreasing features, on the right the top 3 increasing features across the time from year zero, to the maximum age of the dataset are shown.

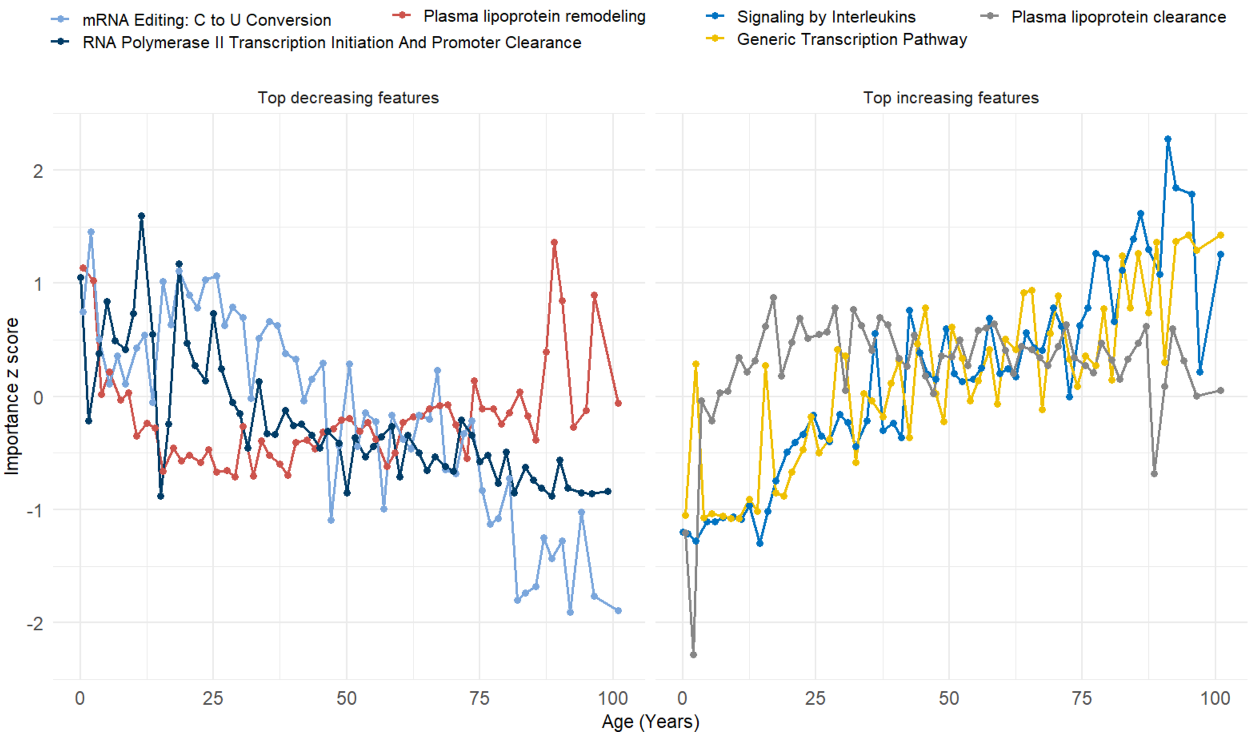

Supplementary figure S4: Visualizing the standardized importance scores across the chronological age on the test set of the pan-tissue dataset, showing only the fourth layer of the network. On the left the top 3 decreasing features, on the right the top 3 increasing features across the time from year zero, to the maximum age of the dataset are shown.

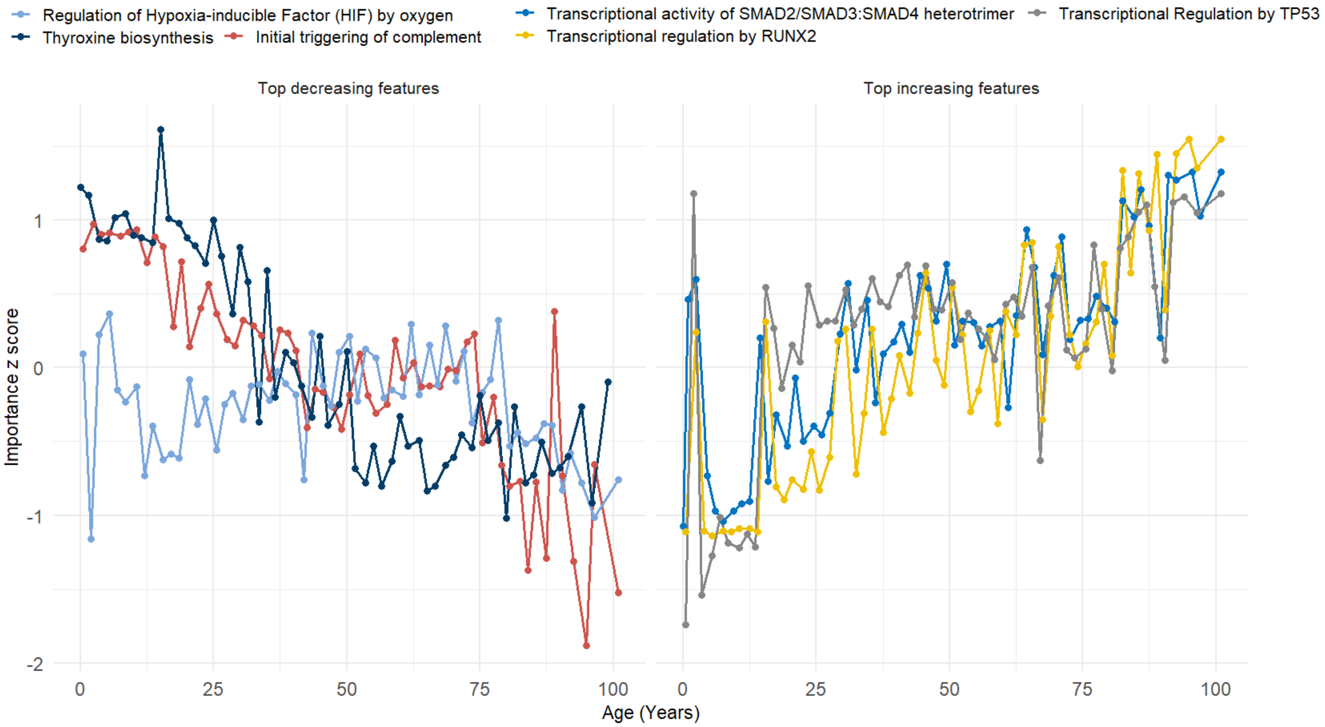

Supplementary figure S5: Visualizing the standardized importance scores across the chronological age on the test set of the pan-tissue dataset, showing only the third layer of the network. On the left the top 3 decreasing features, on the right the top 3 increasing features across the time from year zero, to the maximum age of the dataset are shown.

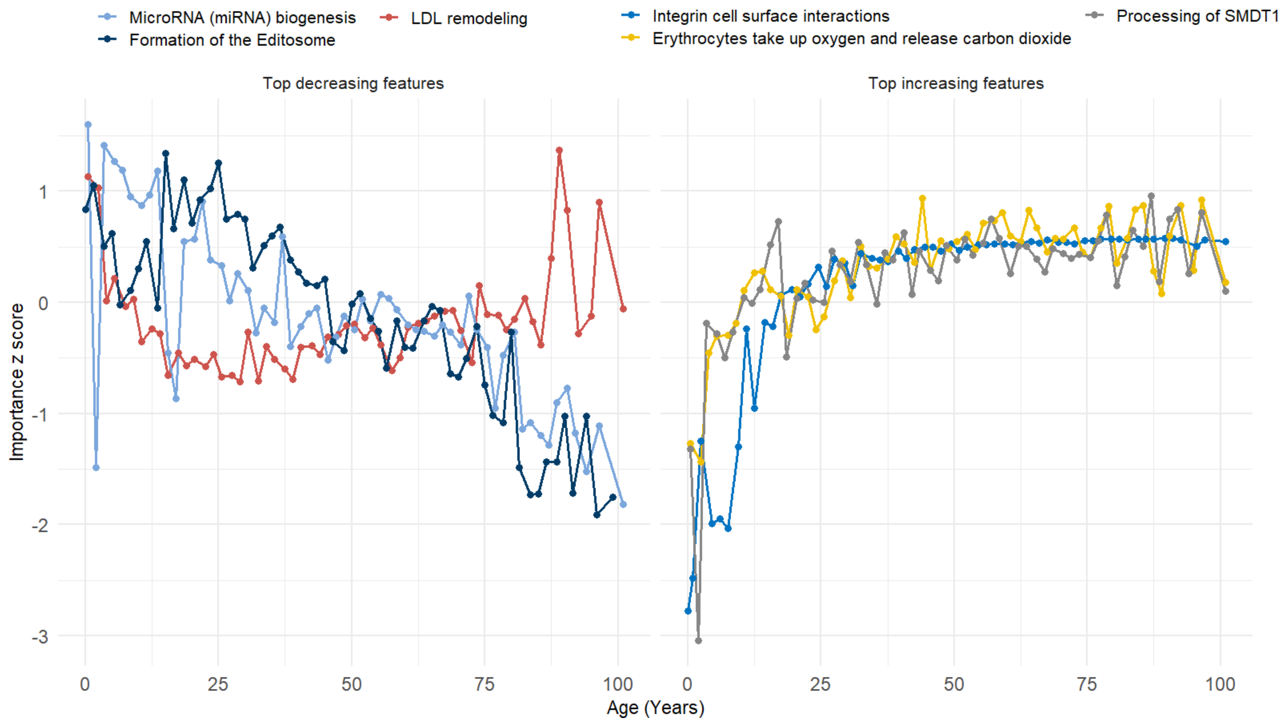

Supplementary figure S6: Visualizing the standardized importance scores across the chronological age on the test set of the pan-tissue dataset, showing only the second layer of the network. On the left the top 3 decreasing features, on the right the top 3 increasing features across the time from year zero, to the maximum age of the dataset are shown.

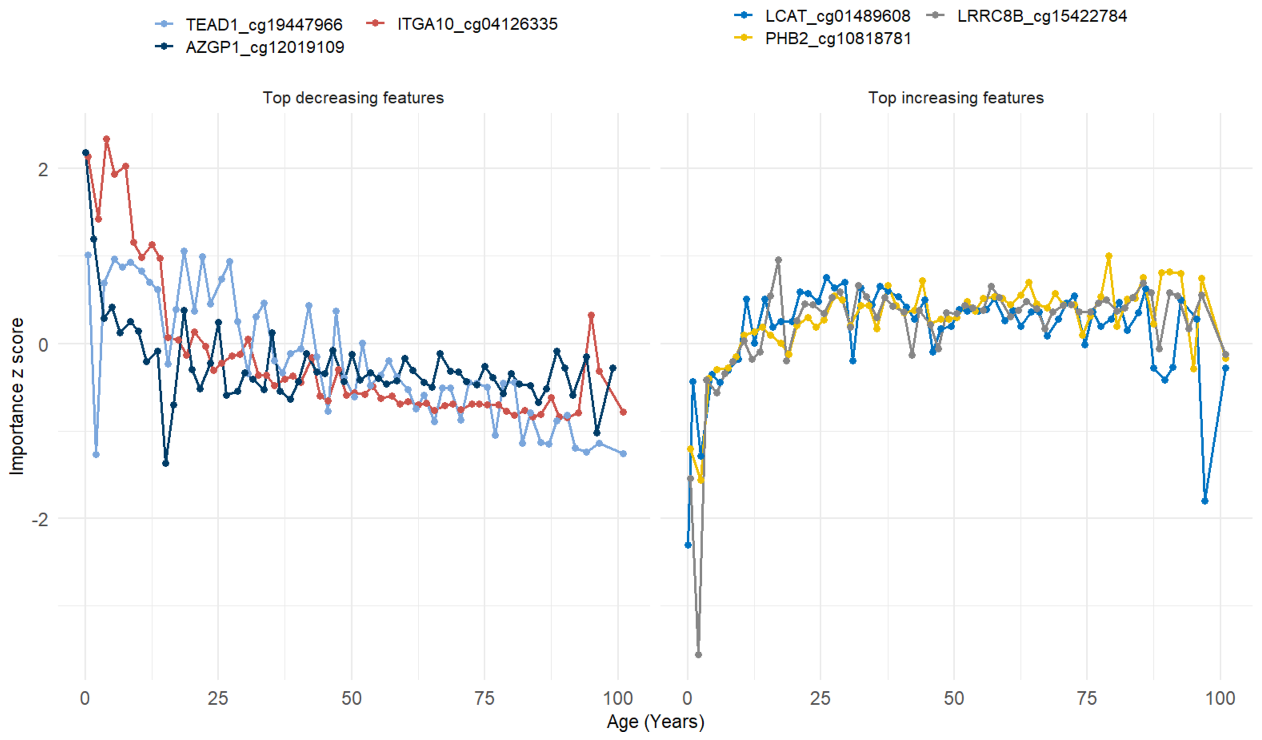

Supplementary figure S7: Visualizing the standardized importance scores across the chronological age on the test set of the pan-tissue dataset, showing only the first layer of the network. On the left the top 3 decreasing features, on the right the top 3 increasing features across the time from year zero, to the maximum age of the dataset are shown.

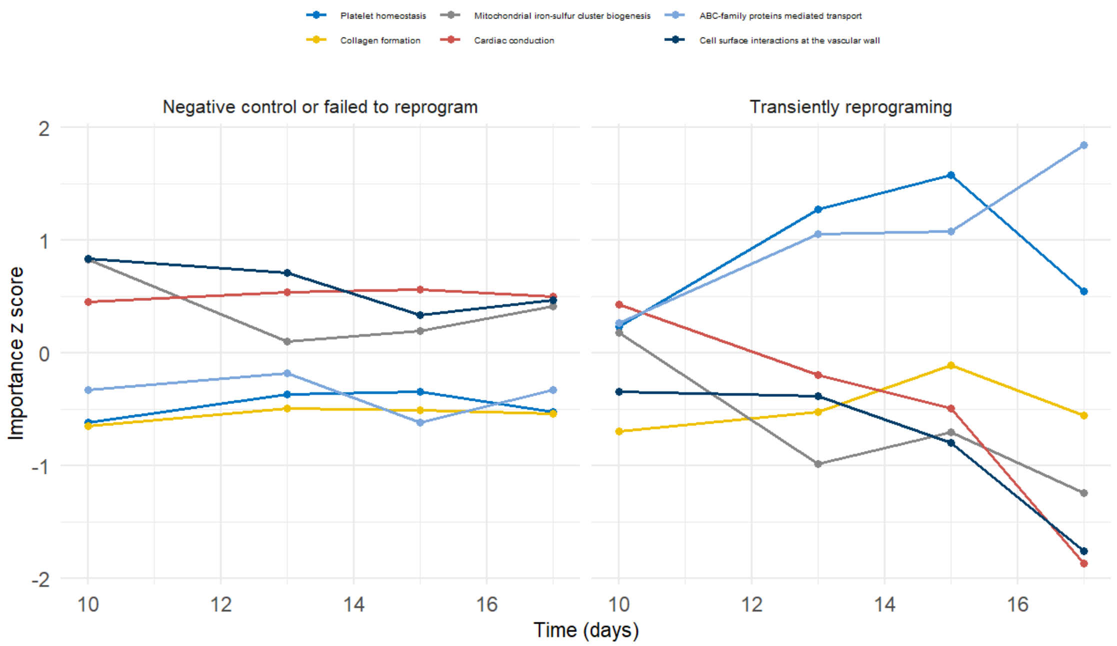

Supplementary figure S8: Visualizing the standardized importance scores across the days measured from the start of the reprogramming, showing only the fifth layer of the network and the most different six features between the negative control, or failed to reprogram group (left) compared to the transiently reprogrammed group (right).

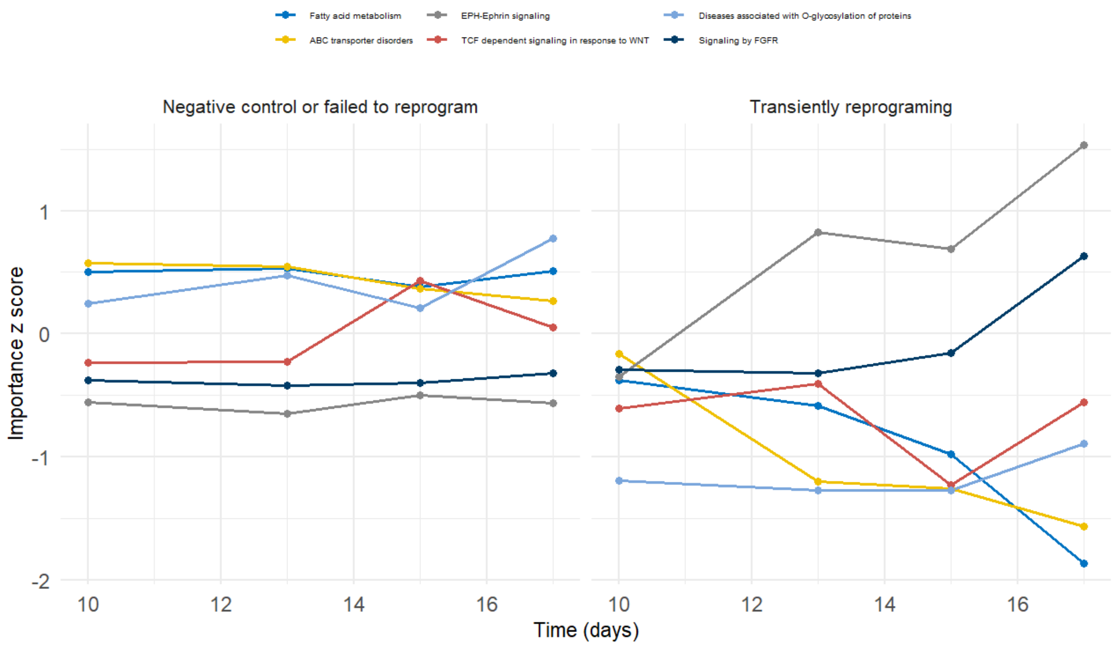

Supplementary figure S9: Visualizing the standardized importance scores across the days measured from the start of the reprogramming, showing only the fourth layer of the network and the most different six features between the negative control, or failed to reprogram group (left) compared to the transiently reprogrammed group (right).

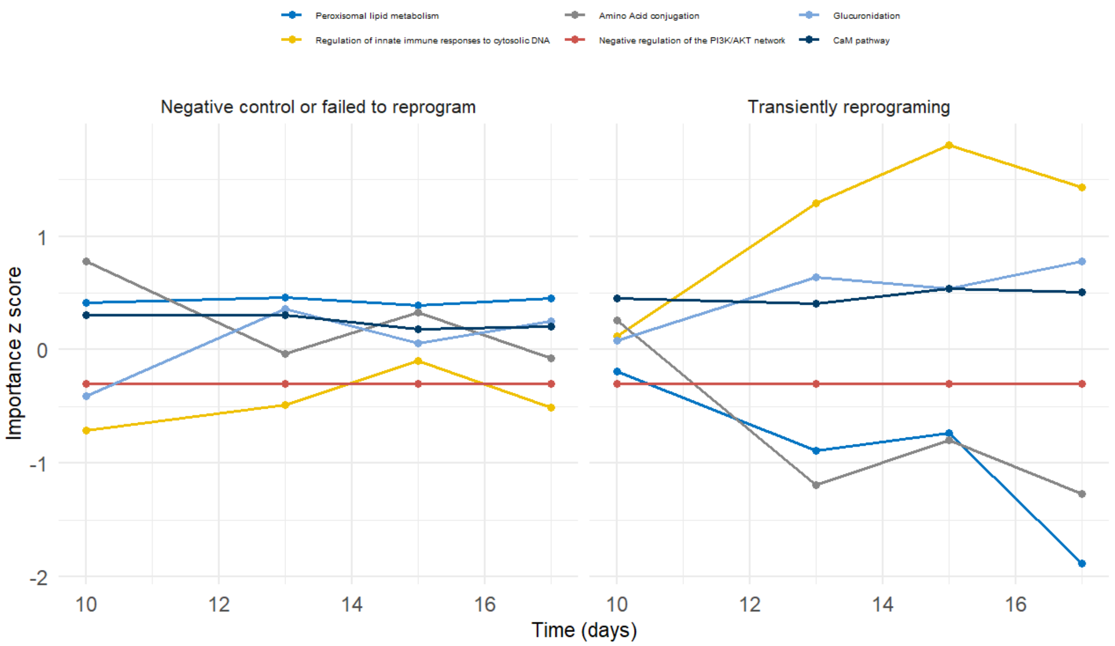

Supplementary figure S10: Visualizing the standardized importance scores across the days measured from the start of the reprogramming, showing only the third layer of the network and the most different six features between the negative control, or failed to reprogram group (left) compared to the transiently reprogrammed group (right).

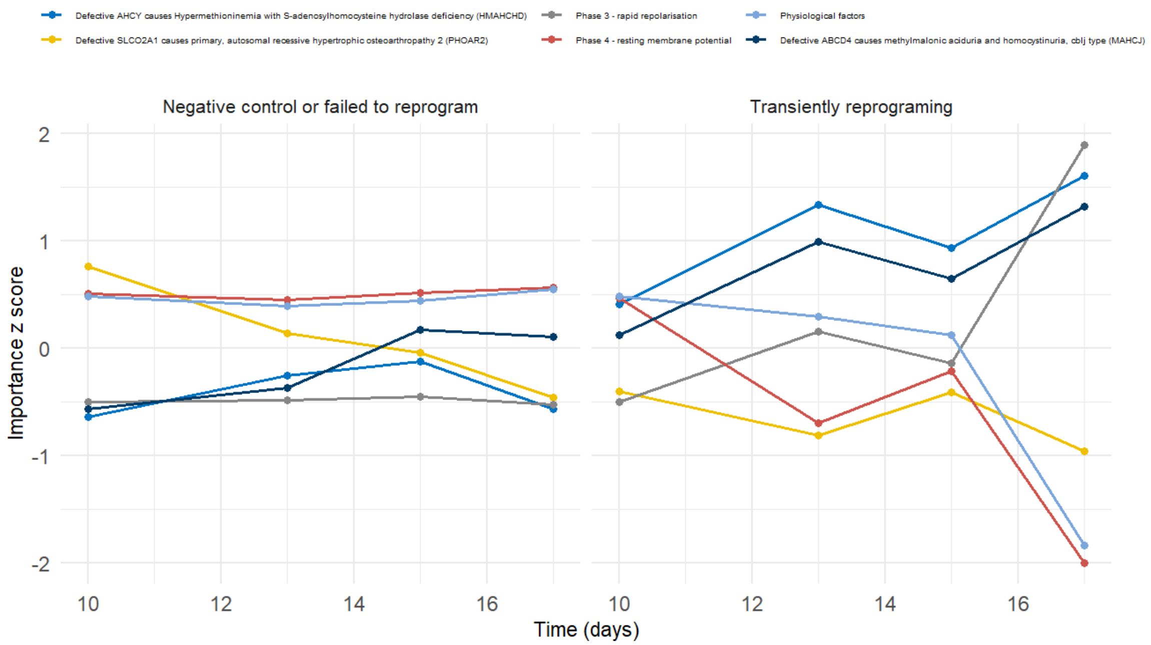

Supplementary figure S11: Visualizing the standardized importance scores across the days measured from the start of the reprogramming, showing only the second layer of the network and the most different six features between the negative control, or failed to reprogram group (left) compared to the transiently reprogrammed group (right).

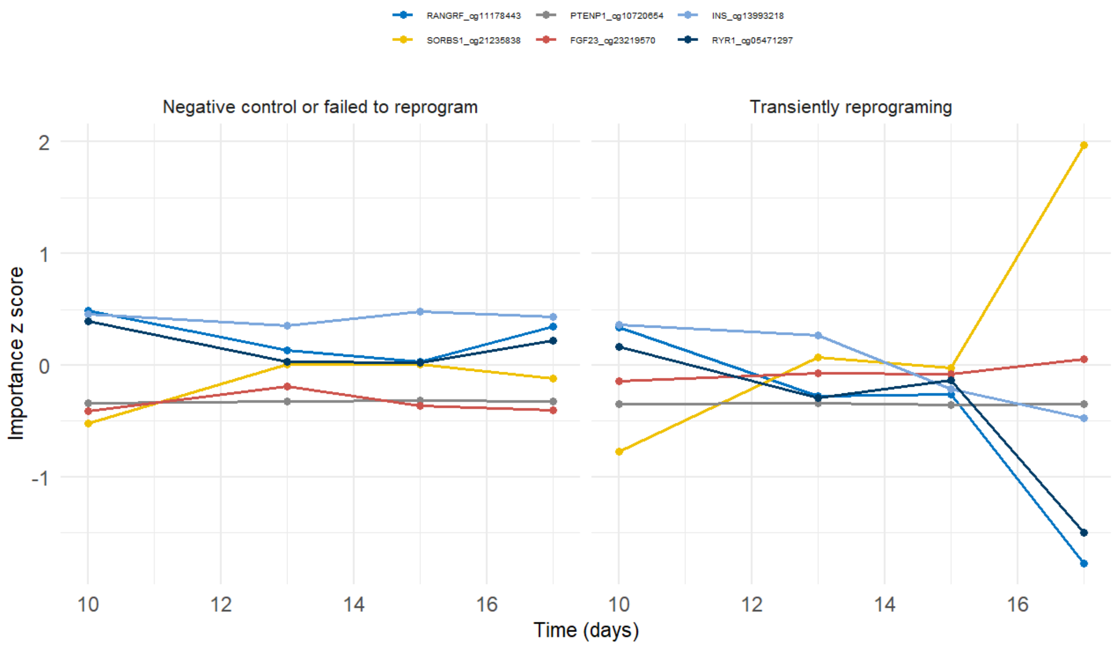

Supplementary figure S12: Visualizing the standardized importance scores across the days measured from the start of the reprogramming, showing only the first layer of the network and the most different six features between the negative control, or failed to reprogram group (left) compared to the transiently reprogrammed group (right).

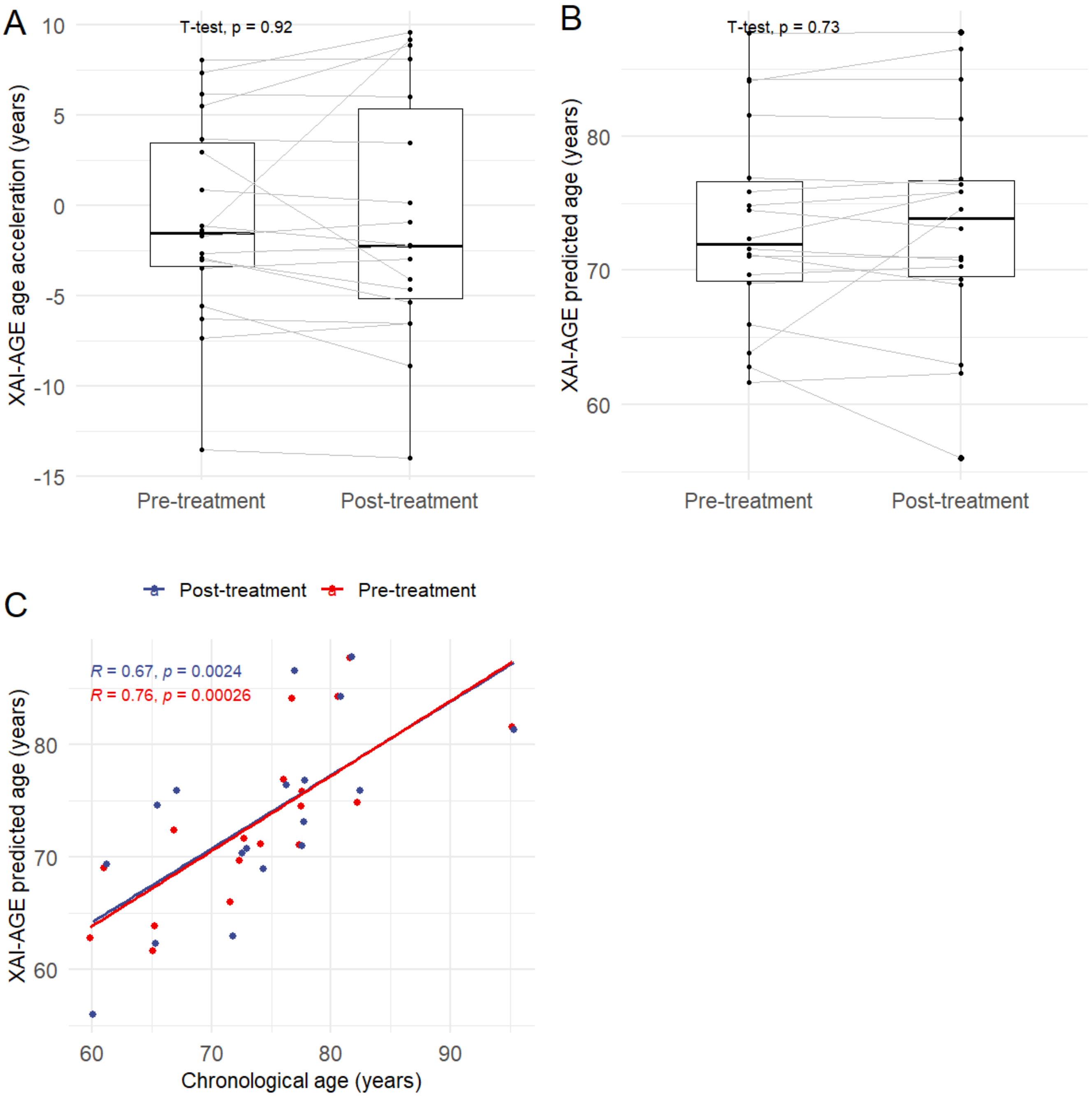

Supplementary figure S13: Box plots (A-B) showing the age acceleration predicted age by XAI-AGE in the umblical cord plasma transfusion dataset. No significant effects were observed between the pre-treatment and post-treatment groups. Scatter plot (C) showing the correlation between the predicted age by XAI-AGE and chronological age in the umblical cord plasma transfusion dataset.

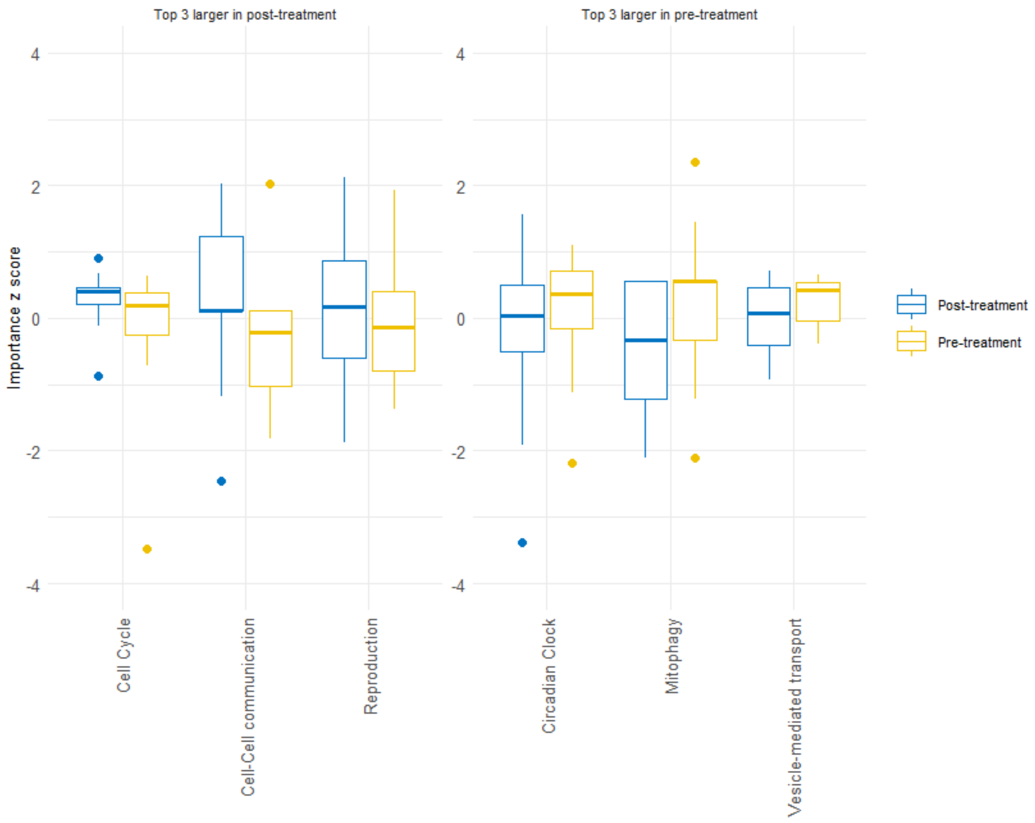

Supplementary figure S14: The six features which either make the largest difference between post-treatment and pre-treatment groups (first three features on the plot), or the largest differences, but between pre-treatment and post-treatment (last three on the plot) in the umbilical cord plasma transfusion dataset from the last layer of the network. No significant effects were observed between the pre-treatment and post-treatment groups.

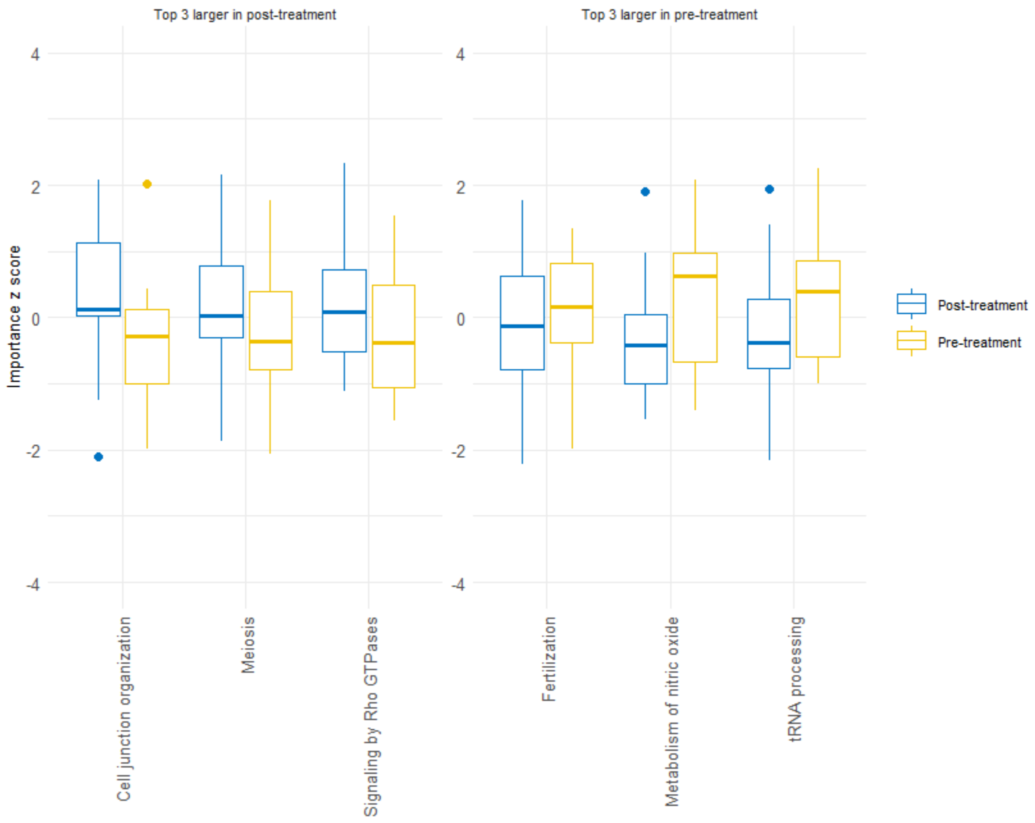

Supplementary figure S15: The six features which either make the largest difference between post-treatment and pre-treatment groups (first three features on the plot), or the largest differences, but between pre-treatment and post-treatment (last three on the plot) in the umbilical cord plasma transfusion dataset from the fifth layer of the network. No significant effects were observed between the pre-treatment and post-treatment groups.

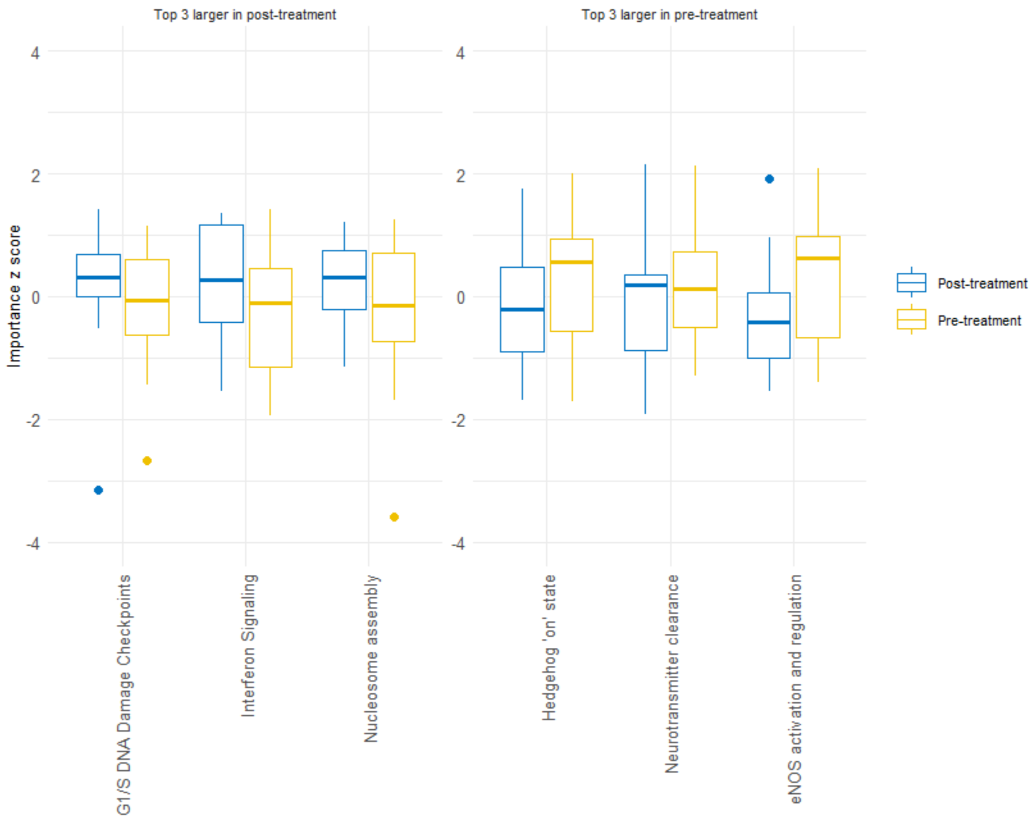

Supplementary figure S16: The six features which either make the largest difference between post-treatment and pre-treatment groups (first three features on the plot), or the largest differences, but between pre-treatment and post-treatment (last three on the plot) in the umbilical cord plasma transfusion dataset from the fourth layer of the network. No significant effects were observed between the pre-treatment and post-treatment groups.

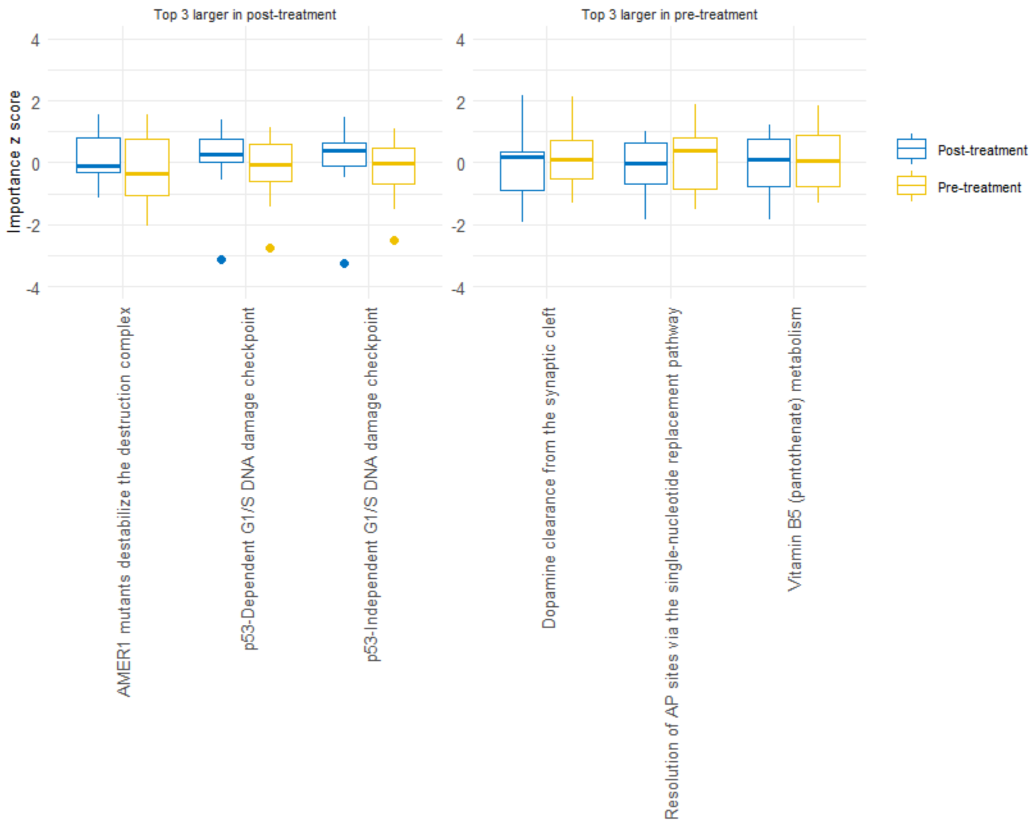

Supplementary figure S17: The six features which either make the largest difference between post-treatment and pre-treatment groups (first three features on the plot), or the largest differences, but between pre-treatment and post-treatment (last three on the plot) in the umbilical cord plasma transfusion dataset from the third layer of the network. No significant effects were observed between the pre-treatment and post-treatment groups.

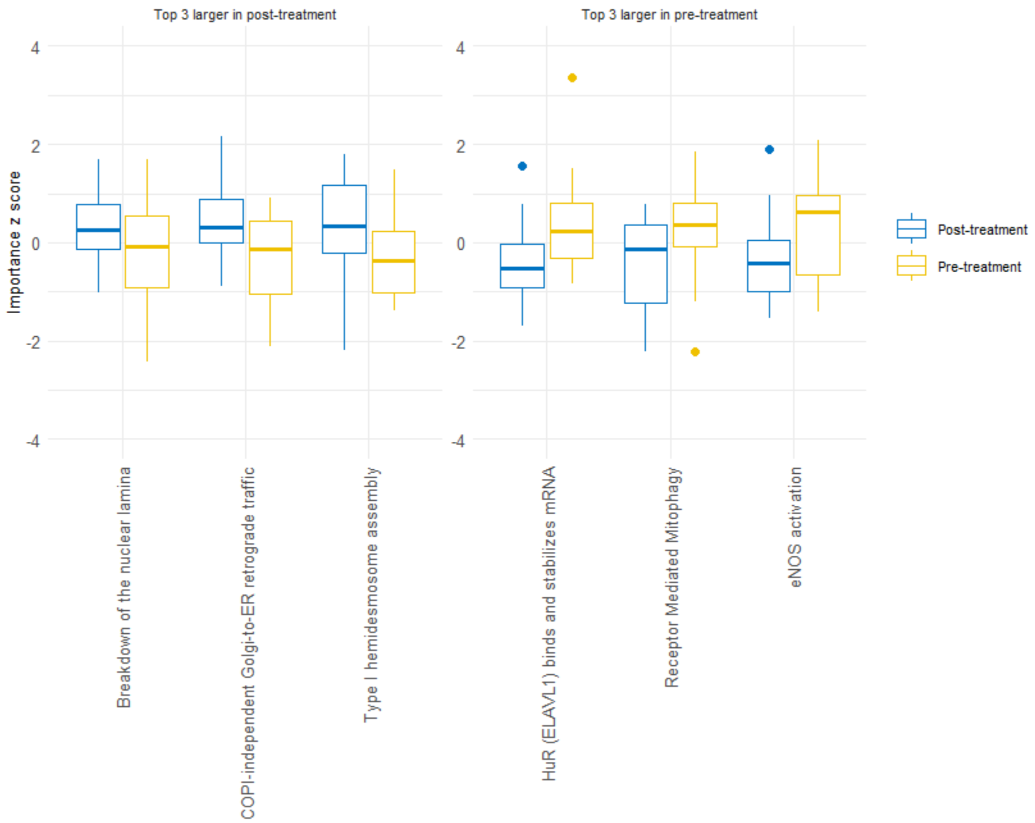

Supplementary figure S18: The six features which either make the largest difference between post-treatment and pre-treatment groups (first three features on the plot), or the largest differences, but between pre-treatment and post-treatment (last three on the plot) in the umbilical cord plasma transfusion dataset from the second layer of the network. No significant effects were observed between the pre-treatment and post-treatment groups.

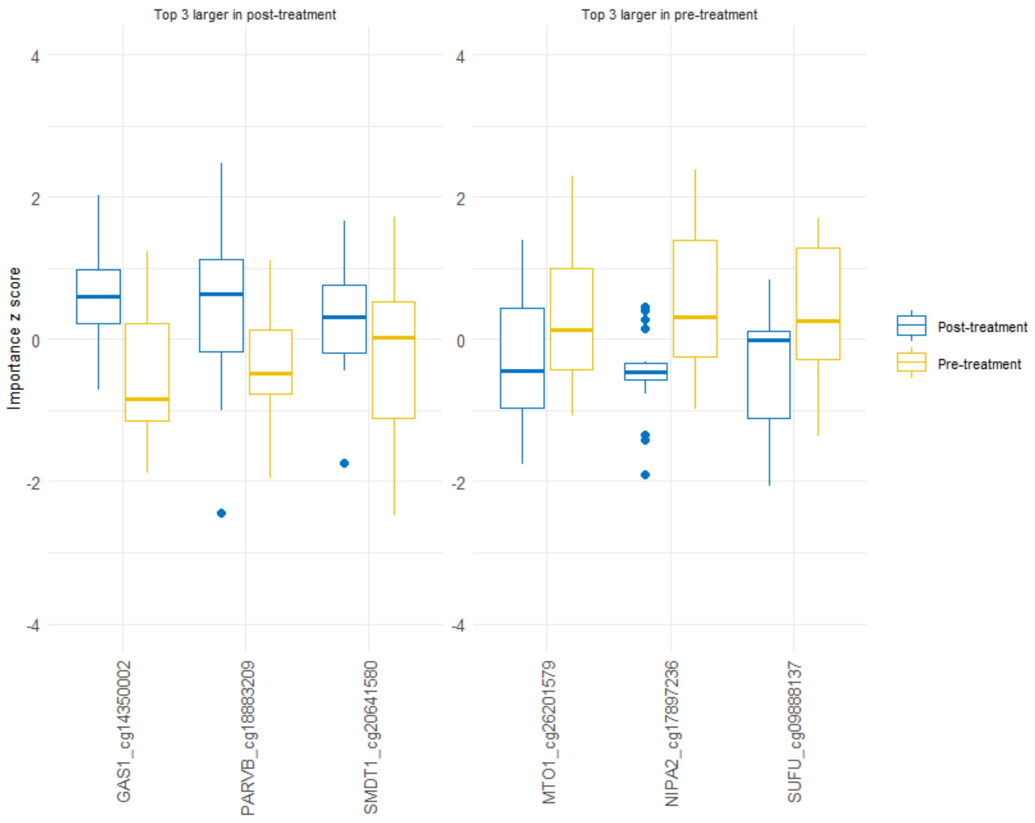

Supplementary figure S19: The six features which either make the largest difference between post-treatment and pre-treatment groups (first three features on the plot), or the largest differences, but between pre-treatment and post-treatment (last three on the plot) in the umbilical cord plasma transfusion dataset from the first layer of the network. No significant effects were observed between the pre-treatment and post-treatment groups.

References

[1] Haitham Elmarakeby, Justin Hwang, Rand Arafeh, Jett Crowdis, Sydney Gang, David Liu, Saud AlDubayan, Keyan Salari, Steven Kregel, Camden Richter, Taylor Arnoff, William Hahn, and Eliezer Van Allen. Biologically informed deep neural network for prostate cancer discovery. *Nature*, 598:1–5, 10 2021.

[2] Martin Aryee, Andrew Jaffe, Hector Corrada Bravo, Christine Ladd-Acosta, Andrew Feinberg, Kasper Hansen, and Rafael Irizarry. Minfi: A flexible and comprehensive bioconductor package for the analysis of infinium dna methylation microarrays. *Bioinformatics (Oxford, England)*, 30, 01 2014.

[3] Avanti Shrikumar, Peyton Greenside, and Anshul Kundaje. Learning important features through propagating activation differences. 04 2017.

[4] Mainak Jas, Titipat Achakulvisut, Aid Idrizović, Daniel Acuna, Matthew Antalek, Vinicius Marques, Tommy Odland, Ravi Garg, Mayank Agrawal, Yu Umegaki, Peter Foley, Hugo Fernandes, Drew Harris, Beibin Li, Olivier Pieters, Scott Otterson, Giovanni De Toni, Chris Rodgers, Eva Dyer, and Pavan Ramkumar. Pyglmnet : Python implementation of elastic-net regularized generalized linear models. *Journal of Open Source Software*, 5, 03 2020.

[5] Ron Edgar and Alex Lash. 6. the gene expression omnibus (geo): A gene expression and hybridization repository. 01 2002.

[6] Zhining Wang, Mark Jensen, and Jean Zenklusen. *A practical guide to The Cancer Genome Atlas (TCGA)*, volume 1418, pages 111–141. 03 2016.

[7] S. Horvath. Dna methylation age of human tissues and cell types. *Genome Biol.*, 14:R115, 2013.
